# Sustainable Approach to Overcome Polylactide Brittleness with Biobased Esters of Isosorbide and Fatty Acids

**DOI:** 10.1021/acssuschemeng.5c01601

**Published:** 2025-05-16

**Authors:** Mario Miranda-Pinzon, Jaume Gomez-Caturla, Juan Ivorra-Martinez, Nestor Guijarro, Xavier Marset, Rafael Balart

**Affiliations:** † Instituto Universitario de Investigación de Tecnología de Materiales (IUITM), Universitat Politècnica de València (UPV), Plaza Ferrándiz y Carbonell 1, 03801 Alcoy, Alicante, Spain; ‡ Instituto de Electroquímica, 16718 Universidad de Alicante, Apdo. 99, E-03080 Alicante, Spain; § Instituto de Síntesis Orgánica (ISO), Universidad de Alicante, Apdo. 99, E-03080 Alicante, Spain

**Keywords:** isosorbide, esterification, fatty acids, plasticizer, polylactide, toughness

## Abstract

This work reports on the design and synthesis of sustainable plasticizers from plant-based isosorbide to enhance the intrinsic brittleness of polylactide (PLA). To keep fully biobased carbon, isosorbide was esterified with fatty acids of varying chain length, leading to isosorbide dibutyrate (IDB), dicaprylate (IDC), and dipalmitate (IDP). These esters were incorporated into PLA at different concentrations. An approach to assess PLA-plasticizer miscibility was conducted by calculating solubility parameters (δ) and the Flory–Huggins interaction parameter, χ. The effect of plasticizer type and concentration on mechanical, thermal, and thermomechanical properties, as well as on microstructure and biodegradation, was also addressed. The results indicated that IDB and IDC notably enhanced PLA toughness, reducing the PLA’s glass transition temperature (*T*_g_) from 60.3 to 27.7 °C with 20 wt % IDC. Consequently, strain at break dramatically increased from 12.8% (PLA) to over 300% with 20 wt % IDB or IDC. In contrast, IDP exhibited limited miscibility, resulting in phase separation, though it still improved the impact strength and ductility. All formulations demonstrated exceptional disintegration in compost soil, underscoring their potential as “double green” plasticizers suitable for PLA. Since both PLA and isosorbide can be industrially derived from starch, this work places starch as a key platform for sustainable polymers.

## Graphical abstract

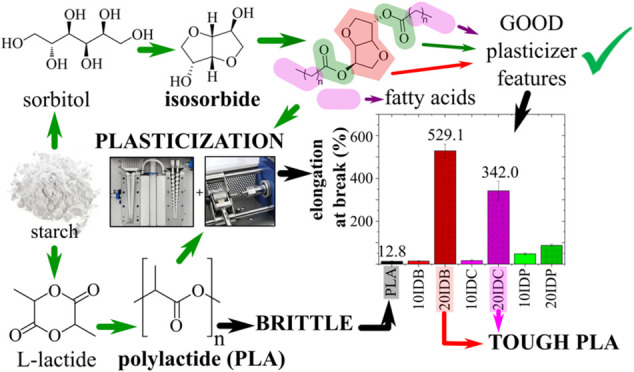

## Introduction

In recent years, social awareness of environmental issues and the need for sustainable development have grown significantly. Within this evolving paradigm, biopolymer development has gained substantial importance and is expected to play a pivotal role in achieving the United Nations’ 2030 Sustainable Development Goals (SDGs).^1^ Among the notable advancements in biopolymer research, materials derived from renewable resources and featuring biodegradability have gained high interest.^2,3^ This category includes polysaccharides such as starch, cellulose, pectin, and chitin,^4,5^ proteins like soybean, gluten, and zein,^6–8^ as well as bacterial polyesters such as polyhydroxyalkanoates (PHA).^9,10^ Starch and other polysaccharides present promising sustainable alternatives to commodity plastics, particularly in the packaging industry.^11,12^ Although starch-based polymers are increasingly used,^13–15^ their properties still fall short of those of commodity plastics.^16^ Regardless of these limitations, starch has emerged as a green platform for polymer and additive synthesis, expanding its potential to produce high-added-value, sustainable polymers.^17,18^ Polylactide (PLA) stands out as one of the most promising for industrial applications,^19^ with its use spanning sectors such as biomedical,^20^ automotive,^21^ electrical/electronics,^22^ and primarily in the packaging industry.^23^ It is, by far, the most cost-effective aliphatic polyester, with an annual production capacity of about 250,000 tons.^24^ Moreover, its widespread use in 3D printing technologies further expands its use as a sustainable alternative to petroleum-derived plastics.^25–27^ PLA offers competitive cost, well-balanced performance, biodegradability, and processing versatility. However, PLA is intrinsically brittle.^28^

Although PLA toughness can be enhanced through copolymerization/cross-linking,^29,30^ and physical blending,^31–33^ plasticization remains the most widely used approach.^34^ Monomeric plasticizers, including citrates,^35^ adipates,^36^ cinnamates,^37^ tartrates,^38^ lactates,^39^ epoxidized vegetable oils,^40^ among others, offer enhanced compatibility and improved toughness, but they tend to migrate over time, leading to aging.^41^ In contrast, oligomeric/polymeric plasticizers, including polyethylene glycol (PEG),^42^ polypropylene glycol (PPG),^43^ and oligomers of lactic acid (OLA),^44^ provide better resistance to migration and aging. However, they show restricted miscibility.^45^ Consequently, PLA plasticization still remains a challenge.

Isosorbide (1,4:3,6-dianhydro-d-glucitol) is a bicyclic compound industrially obtained through the hydrogenation of glucose followed by double dehydration. Glucose is primarily obtained from the hydrolysis of starch.^46^ Isosorbide is a promising biobased diol with broad applications in pharmaceuticals, surfactants, polymer plasticization, and catalysis.^46^ This nontoxic, biodegradable, chiral, and rigid molecule features a unique chemical structure suitable for use in the polymer industry.^47^ Isosorbide diesters exhibit structural similarities to conventional phthalate-based plasticizers for PVC.^48^ According to Bocqué et al., an ideal plasticizer should include aliphatic chains (acting as spacers), ester groups (providing cohesion), and aromatic ring (enhancing compatibility).^49^ Although isosorbide lacks an aromatic structure, its heterocyclic nature and polar characteristics contribute to excellent compatibility with the polymer. Previous studies have highlighted the exceptional plasticization performance of isosorbide dioctanoate in PLA processed via thermocompression.^50^ Similarly, isosorbide dicaprylate has been proposed as a secondary plasticizer for PVC,^51^ while recent research has explored the influence of alkyl chain length on the plasticization efficiency of some isosorbide diesters on PVC.^52^

Considering that both PLA and isosorbide are industrially derived from starch, it is important to bear in mind the steady growth of the global starch market. The food and beverage industry remains the primary consumer, accounting for 55% of the global market. The remaining 45% is used in nonfood (human) applications, including animal feeding, pharmaceuticals, paper and textile industries, and packaging, among others. In 2020, global starch production reached 119.6 million tons, with projections indicating a compound annual growth rate (CAGR) of 5.0%, reaching 160.3 million tons by 2026. In this scenario, the increasing use of starch in various industries, including food and nonfood applications, is likely to be a key driver for this extraordinary growth.^53^ This study opens new possibilities for the development of cost-effective and sustainable plastics with improved properties, following an environmentally friendly route based on starch for nonfood applications as raw material. Additionally, this research gives an in-depth view on the effect of the alkyl chain length of the fatty acids used in the esterification of isosorbide to offer “double green” plasticizers for PLA, focusing on the usefulness of some indicators of miscibility, namely, the solubility parameter (δ), Flory–Huggins interaction parameter (χ), as well as the overall mechanical, thermal, thermomechanical, morphological, and compost biodegradation properties of PLA materials plasticized with isosorbide diesters.

## Experimental Section

### Materials

A commercial-grade polylactide (PLA) PURAPOL L130 was obtained from Total Corbion PLA. d-Isosorbide (>98% purity), butyryl chloride (>99%), capryloyl chloride (>99%), and palmitoyl chloride (>98%) were supplied by Sigma-Aldrich (Madrid, Spain). All solvents and reagents were obtained from commercial sources and used without further purification.

### Synthesis of Isosorbide Diesters

This procedure (A) was adapted from a literature report.^50^ Isosorbide (80 mmol, 11.8 g) was charged in a two-neck round-bottom flask equipped with a magnetic stirring bar and a condenser. The system was evacuated under vacuum and backfilled with argon (×3). Dry dichloromethane (125 mL) and triethylamine (240 mmol, 33.8 mL) were then added, and the mixture was cooled in an ice bath. Subsequently, the corresponding acid chloride (160 mmol) was added dropwise at 0 °C. After addition, the mixture was stirred at room temperature for 2 h and then heated to 45 °C for 30 min. After cooling to room temperature, the reaction mixture was sequentially washed with water and brine (×2). The organic layer was dried over MgSO_4_, filtered, and concentrated *in vacuo* to yield the crude product. Further purification was achieved by column chromatography over silica gel using mixtures of hexanes and ethyl acetate as an eluent.

### Manufacturing of PLA Blends with Isosorbide Diesters

PLA was dried at 40 °C for 24 h. Plasticized PLA formulations were prepared by incorporating 10 and 20 wt % of each synthesized isosorbide diester, followed by extrusion and injection molding. The sample code includes a number (wt % plasticizer) and the abbreviation of the respective isosorbide ester: IDB, IDC, and IDP, for isosorbide dibutyrate, dicaprylate, and dipalmitate, respectively. The blends were obtained using a DSM Xplore MC 15 twin-screw microcompounder from Xplore Instruments BV at 100 rpm. Mixing was performed at 180 °C for 2 min. Standardized specimens were obtained in a micro injection molding unit DSM Xplore IM12 from Xplore Instruments BV. The injection molding was carried out at 190 °C under a pressure of 8 bar.

### Fourier Transform Infrared Spectroscopy (FTIR)

FTIR characterization was carried out in a Bruker S.A. Vector 22 spectrometer equipped with a PIKE MIRacle single reflection diamond ATR accessory. FTIR analysis was performed on the samples with 20 scans, a spectral resolution of 4 cm^–1^, and a wavelength range from 4000 to 600 cm^–1^.

### Characterization of the Synthesized Isosorbide Diesters

^1^H NMR (400 MHz) and ^13^C NMR (101 MHz) spectra were acquired using a BRUKER AV400 spectrometer under proton-coupled mode and proton-decoupled mode, respectively, at 20 °C. Chemical shifts (δ) are reported in parts per million, while coupling constants (*J*) are reported in hertz. Low-resolution mass spectra (EI) were recorded at 70 eV on an Agilent Technologies GC/MS-8890N, with fragment ions reported in mass-to-charge (*m*/*z*) ratio alongside their relative intensities (%). High-resolution mass spectra (EI) were obtained at 70 eV using an Agilent 7200 quadrupole time-of-flight (Q-TOF) spectrometer equipped with a Direct Insertion Probe (73DIP-1). Infrared spectra were collected in a Jasco FT/IR-4100 Fourier transform infrared spectrometer. Thin-layer chromatography (TLC) was carried out on Schleicher & Schuell F1400/LS 254 plates coated with a 0.2 mm layer of silica gel; detection by UV_254_ light. Column chromatography was performed using silica gel 60 (40–63 mesh).

### Thermal Properties

Differential scanning calorimetry (DSC) measurements were carried out in a Discovery DSC 25 calorimeter from TA Instruments under a nitrogen flow (66 mL/min). Samples underwent a 3-step thermal program: heating from 30 to 180 °C; cooling to 0 °C; heating from 0 to 220 °C (heating/cooling rate of 10 °C/min). Thermal degradation was evaluated through thermogravimetric analysis (TGA) using a TG-DSC2 thermobalance from Mettler Toledo. Specimens were heated from 30 to 700 °C at 10 °C/min under air atmosphere. Dynamic mechanical thermal analysis (DMTA) was carried out in a Mettler Toledo DMA1 in a single cantilever, with a frequency of 1 Hz, and a cantilever deflection amplitude of 10 μm. Rectangular specimens measuring 25 × 10 × 4 mm^3^ were subjected to heating from −50 to 100 °C, at 2 °C/min.

### X-ray Diffraction Spectroscopy (XRD)

X-ray diffraction (XRD) patterns were acquired with a KRISTALLOFLEX K 760-80F X-ray generator operating at 40 kV and 40 mA. The Cu Kα radiation (λ = 0.154 nm) was nickel-filtered to enhance the spectral purity. Diffraction data were collected over a scattering angle range (2θ) from 5 to 70° with a step size of 0.05° and a scanning speed of 1 °/min. XRD characterization was carried out on samples with dimensions 10 × 10 × 2 mm^3^.

### Mechanical Properties

The tensile values were evaluated using a Duotrac-10/1200 universal testing machine from SAE Ibertest, at room temperature with a 5 kN load cell and a crosshead speed of 10 mm/min following ISO 527. Impact resistance was assessed through Charpy impact testing on notched specimens with a “V”-type notch (radius: 0.5 mm) using a 6-J pendulum impact tester from Metrotec S.A. following ISO 179. At least five different specimens per formulation were tested in all mechanical characterizations.

### Morphology

The morphology of PLA blends was analyzed by field emission scanning electron microscopy (FESEM) in a ZEISS ULTRA 55 microscope from Oxford Instruments operated at 2 kV and a working distance of 4 mm. Prior to FESEM examination, the specimens underwent a sputter-coating process with a gold–palladium alloy sputter coater from Quorum Technologies, Ltd.

### Compost Disintegration

The disintegration under composting conditions was assessed following ISO 20200. Rectangular specimens (10 × 10 × 2 mm^3^) were prepared, dried at 40 °C for 24 h, weighed, and subsequently buried in a bioreactor (30 × 20 × 10 cm^3^), containing a synthetic compost soil. The samples were subjected to aerobic degradation conditions at 58 °C in an air-circulating oven for 6 weeks. At weekly intervals, specimens were retrieved from the bioreactor, rinsed with distilled water, and reweighed to monitor the mass loss as an indicator of disintegration.

## Results and Discussion

### Spectroscopic Characterization of the Synthesized Isosorbide Diesters and Their Blends with PLA

Figure 1 shows the ^1^H NMR spectra of the synthesized isosorbide diesters, while their ^13^C NMR spectra are shown in Figures S1–S3.

**Figure 1:**
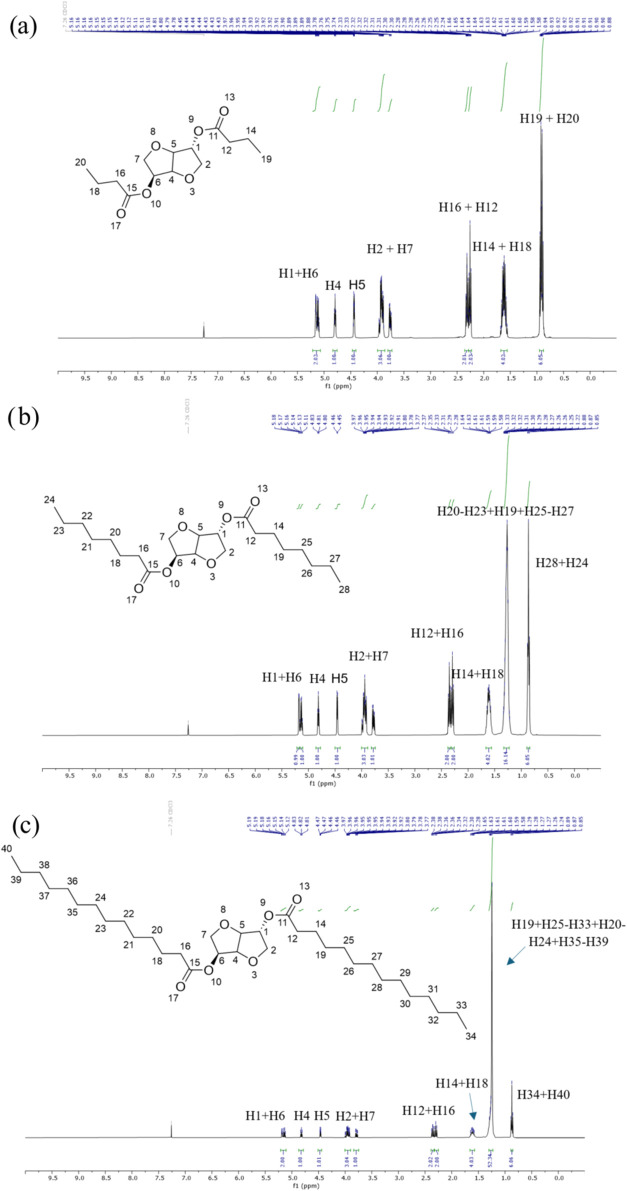
^1^H NMR (400 MHz, CDCl_3_) of the synthesized isosorbide diesters: (a) isosorbide-2,5-dibutyrate (IDB), (b) isosorbide-2,5-dicaprylate (IDC), and (c) isosorbide-2,5-dipalmitate (IDP).

Isosorbide-2,5-dibutyrate (IDB) and isosorbide-2,5-dicaprylate (IDC) were yellow oils, while isosorbide-2,5-dipalmitate (IDP) was obtained as a white solid with a melting point of 68–69 °C (see the Supporting Information for detailed physical and spectroscopic characterization). Moreover, the FTIR characterization of neat PLA and its blends with isosorbide diesters is shown in Figure S4.

### An Approach to Miscibility of PLA and Isosorbide Diesters by the Solubility Parameters and the Flory–Huggins Interaction Parameter

Table S1 gathers the solubility parameters (δ), along with their dispersive-δ_d_, polar-δ_p_, and hydrogen bonding-δ_H_ contributions, for PLA and the different synthesized isosorbide diesters, calculated using the Van Krevelen & Hoftyzer method.^54^ Moreover, Table S2 shows the solubility parameters of some common PL solvents. The molar volumes were also estimated by the group contribution method proposed by Fedors and Hoy.^55,56^ The solubility region of PLA can be graphically represented by a spherical region in a 3D space with the axes δ_d_, δ_p_, and δ_H_. This spherical region has center coordinates located at δ_d_PLA_, δ_p_PLA_, and δ_H_PLA_, and a radius, *R*_0_, with a value of 10.7 MPa^1/2^, for PLA.^57^ The solubility of a plasticizer with PLA can be graphically assessed by plotting its δ_plast_ coordinates (δ_d_plast_, δ_p_plast_, and δ_H_plast_). If these coordinates fall into the solubility region of PLA, then good miscibility may be expected. If the δ_plast_ coordinates fall out of the solubility region of PLA, then poor miscibility could be expected. This can also be numerically obtained by determining the distance between PLA and the corresponding plasticizer, *R*_a_, as indicated in eq 1. The constant 4 in the first term, related to the dispersive contribution, δ_d_ is widely used to obtain spherical solubility regions instead of spheroidal regions, which favors plot representations.

1 $$R_{a}=\sqrt{{4\left(\delta_{\mathrm{d\_PLA}}-\delta_{\mathrm{d\_plast}}\right)}^{2}+{\left(\delta_{\mathrm{p\_PLA}}-\delta_{\mathrm{p\_plast}}\right)}^{2}+{\left(\delta_{\mathrm{H\_PLA}}-\delta_{\mathrm{H\_plast}}\right)}^{2}}$$

An approximate indicator of miscibility is the relative energy difference (RED), which is the *R*_a_-to-*R*_0_ ratio, as shown in eq 2. If RED < 1 (δ_plast_ inside the solubility region of PLA), then good miscibility is to be expected, and the lower the value of RED, the higher the miscibility. On the contrary, if RED > 1 (δ_plast_ out of the solubility region of PLA), then poor miscibility will be expected. If RED = 1, the solubility is in the threshold.

2 $$\mathrm{RED}=\frac{R_{a}}{R_{0}}$$

As expected, all synthesized isosorbide diesters exhibit a RED value below 1. This is an approximate indicator of good miscibility with PLA.^57^ As expected, since IDB has the lowest molecular weight (286.324 g/mol), it provides δ_d_ and δ_p_ values that are closely aligned to those of PLA, resulting in a small distance, *R*_a_. Table S1 compiles the solubility parameters of commonly used PLA plasticizers. Additionally, Table S2 presents the solubility parameters of some widely used PLA solvents taken from Hansen.^58^

The PLA-IDB blend exhibits a RED value of 0.48, identical with that of the widely used triethyl citrate (TEC) plasticizer, which is known for its high miscibility with PLA. As the alkyl chain length increases, the RED also rises. PLA-IDC blend presents a RED value of 0.68, which is still similar to citrates, adipates, and polyethylene glycol (PEG), while the PLA-IDP blend exhibits a RED value of 0.85, similar to epoxidized soybean oil (ESO) (0.82). Since phase separation has been reported in plasticized polyesters with ESO,^59^ phase separation in PLA-IDP blend could occur too. As the fatty acid chain length increases, the number of hydrophobic −CH_2_– moieties increases, resulting in higher dispersive contribution, δ_d_, while the polar, δ_p_, and hydrogen bonding, δ_H_, contributions are remarkably reduced. This progressive increase in hydrophobicity results in larger *R*_a_ values and, subsequently, higher RED values. IDP (with the highest molecular weight) exhibits the highest RED value of 0.85. Although still below 1, this relatively elevated value could indicate limited miscibility with PLA. Figure 2 depicts the Bagley’s solubility region of PLA with the synthesized diesters and some common PLA plasticizers (see Figure S5 regarding the Bagley’s solubility region of PLA and common PLA solvents, for comparison). It is noteworthy that IDB is close to acetyltributyl citrate (ATBC), and tributyrin, both of which have been widely employed in PLA plasticization. Similarly, IDC is close to dibutyl adipate (DBA) and dibutyl phthalate (DBP), which have also been proposed as effective PLA plasticizers. In contrast, IDP falls inside the solubility region of PLA, but it is located close to epoxidized soybean oil (ESO), bis­(2-ethylhexyl) adipate (DEHA), and bis­(2-ethylhexyl) phthalate (DEHP), all of which have demonstrated limited miscibility with PLA.

**Figure 2:**
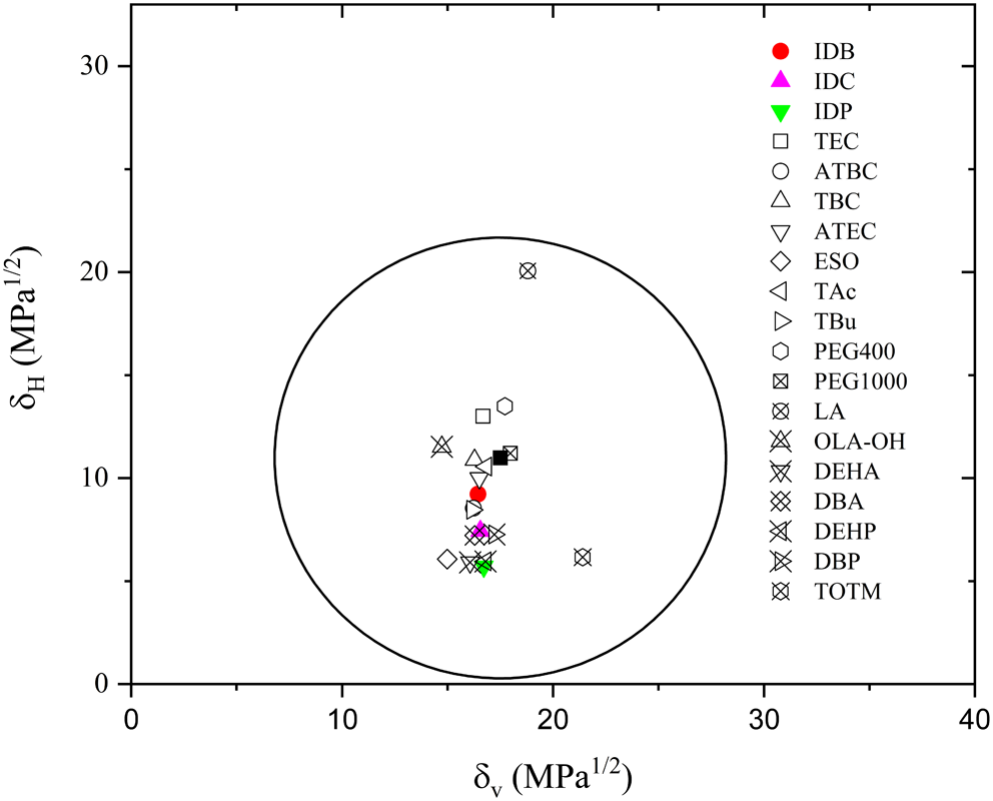
Bagley’s solubility diagram for polylactide and the synthesized isosorbide diesters. The plot also includes the information on some common PLA plasticizers.

The critical interaction parameter χ_crit_ was calculated for PLA considering its molecular weight, yielding a value of χ_crit_ = 0.52 according to the Flory–Huggins theory (see the Supporting Information). As the χ interaction parameter increases, the miscibility decreases and, depending on the solvent (plasticizer) and temperature, phase separation may occur. Such high values for χ above 3 have been reported in full immiscible systems.^60^ The χ interaction parameter was found to be χ = 0.65 for IDB at room temperature, which is close to the threshold value of χ_crit_ = 0.52, further confirming good miscibility.^61^ IDC has a χ = 1.26 at room temperature, which indicates that phase separation may occur at high plasticizer concentrations. Regarding IDP, with the highest molecular weight, its χ = 2.57 at room temperature, thus indicating a strong tendency for phase separation, even at low plasticizer concentrations.

### Thermal Properties

PLA exhibits a glass transition temperature (*T*_g_) of 60.3 °C (Figure 3). It also shows two crystallization processes before melting.^62^ The first corresponds to the cold crystallization peak (*T*_cc_) at 104.8 °C, while the second stands for a premelting crystallization peak (*T*_pmc_) at 159.9 °C, which is related to the α′ to α crystalline phase transition.^63^ The melting peak temperature (*T*_m_) of PLA is observed at 174.4 °C (Table 1), typical for semicrystalline PLA.^64^ The plasticization efficiency is usually assessed as the ability of a plasticizer to depress the glass transition temperature.^45^ In this study, the effect of the fatty acid chain length is particularly noteworthy. As summarized in Table 1, plasticized PLA with 10 wt % IDB exhibits a noticeable decrease in *T*_g_ down to 47.8 °C, confirming its plasticization efficiency. As expected, this decrease in *T*_g_ is even more pronounced in PLA/20IDB with a *T*_g_ of 36.9 °C. It is noteworthy that PLA/20IDC shows the lowest value of 27.7 °C, which is even lower than the value reported by Yang et al.^50^ This confirms that the processing technique plays a crucial role in plasticization effectiveness. Conversely, plasticization with IDP is less effective as DSC reveals a more limited *T*_g_ decrease down to 56–57 °C. Similar findings were reported by Ferri et al.^65^ in plasticized PLA with maleinized linseed oil, and epoxidized vegetable oils.^66^ DSC thermograms of plasticized PLA with IDP exhibit an additional melting peak at 69.4 °C, corresponding to the melting of IDP crystalline domains. This confirms the limited miscibility of IDP within the PLA matrix.

**Figure 3:**
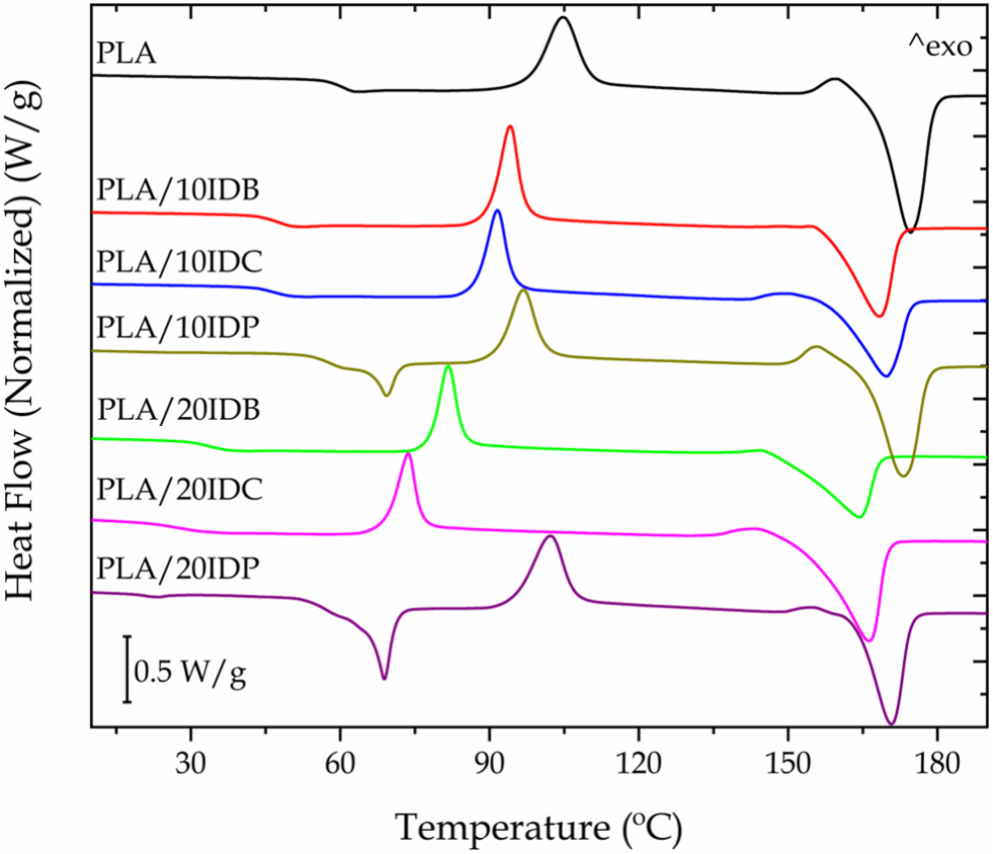
DSC of the second heating cycle of neat PLA and plasticized PLA formulations with varying isosorbide diester type and content.

**Table 1:** Main Thermal Parameters of Neat PLA and Plasticized PLA Formulations with Varying Isosorbide Diester Type and Content Obtained by DSC and TGA

	DSC thermal parameters								TGA thermal parameters		
code	*T*_g_ (°C)	*T*_cc_ (°C)	Δ*H*_cc_ (J/g)	*T*_pmc_ (°C)	Δ*H*_pm_ (J/g)	*T*_m_ (°C)	Δ*H*_m_ (J/g)	% crystallinity	*T*_5_ (°C)	*T*_max_ (°C)	*T*_95_ (°C)
PLA	60.3	104.8	25.1	159.9	4.8	174.4	39.2	9.9	332.6	382.2	392.5
PLA/10IDB	47.8	94.1	23.2			168.4	34.0	12.8	255.3	383.2	394.4
PLA/10IDC	47.2	91.6	20.9	150.3	3.2	169.8	34.2	12.0	314.2	384.2	393.5
PLA/10IDP	57.6	96.8	21.1	155.7	3.9	165.7	35.4	12.3	319.7	374.8	385.6
PLA/20IDB	36.9	81.6	16.5			164.3	30.1	18.2	240.7	385.6	394.3
PLA/20IDC	27.7	73.7	15.9	143.1	1.6	166.2	30.9	17.9	294.7	384.3	391.6
PLA/20IDP	56.2	102.2	23.0	154.8	1.7	170.7	33.0	11.1	306.7	376.1	386.3

As plasticization enhances chain mobility, the crystallization peak temperatures (*T*_cc_, *T*_pmc_) shift toward lower values. Among the synthesized plasticizers, the most significant reduction in both *T*_cc_ and *T*_pmc_ is observed in PLA/20IDC. This effect may be attributed to the higher nucleating ability of the plasticized PLA formulations compared to unplasticized PLA.^67^ Moreover, as shown by DSC, most of the crystallinity of PLA is developed during the cold crystallization, thus indicating very low crystallization during cooling from the melt. As seen in Table 1, all formulations show low crystallinity (between 9 and 12%), while plasticized PLA with 20 wt % IDB and 20 wt % IDC show slightly higher crystallinity values of around 18.0%. This indicates that all formulations are mainly amorphous after processing. These findings agree with those reported by Simmons et al.,^68^ who observed very low crystallinity on PLA of 5% after compression molding. These results were further confirmed by XRD characterization (Figure S6 and Discussion section).

Regarding thermal degradation, PLA degrades under a single-step process (Figure S7), characterized by an onset (temperature at which a 5 wt % mass loss occurs, or *T*_5_) of 332.6 °C, and a maximum degradation rate temperature (*T*_max_) of 382.2 °C. The thermal degradation of PLA plasticized with isosorbide diesters is strongly influenced by the fatty acid chain length. The molecular weight of the synthesized isosorbide esters follows this trend: IDB (286.32 g/mol) < IDC (398.53 g/mol) < IDP (622.96 g/mol). Given that lower-molecular-weight compounds are generally more volatile than high-molecular-weight compounds, the fatty acid chain length significantly affects the main thermal degradation parameters. This is important since at moderate temperatures, this phenomenon could lead to plasticizer removal during processing, ultimately reducing its efficiency. As shown in Table 1, *T*_5_ for PLA plasticized with 10 and 20 wt % IDB reaches the lowest values of 255.3 and 240.7 °C, respectively. In contrast, the *T*_5_ for IDC and IDP-plasticized PLA formulations is higher, as expected. These findings are very important since they mean the plasticizer is not lost during processing at 190 °C, which is the recommended processing temperature for this PLA commercial grade in both extrusion and injection molding processes. Llanes et al.^69^ observed that in PLA plasticized with dibutyl maleate (DBM) and dibutyl fumarate (DBF), some degree of plasticizer volatilization occurs during processing (ranging from 14.3 to 29.0%) due to their low molecular weights. However, the plasticization efficiency was maintained. Therefore, TGA results further confirm no plasticizer loss during processing. Regarding *T*_max_, since it is primarily associated with the thermal degradation of the PLA backbone, no significant changes are observed.

### Mechanical and Thermomechanical Properties

Figure 4 summarizes the main mechanical properties, as well as the visual appearance of the specimens after the tensile test, showing the exceptional plasticization isosorbide diesters provide to PLA. PLA exhibits a tensile strength σ_B_ of 67.7 MPa, but has a relatively low elongation at break, ε_B_ (12.8%), which accounts for its intrinsic brittleness. The addition of 10 wt % IDB or 10 wt % IDC does not result in a significant improvement in ductile properties, although σ_B_ decreases by 15–17 MPa (Figure 4a). It has been reported that the brittle–ductile transition needs a certain threshold to be triggered and become effective. Although plasticization effects were evident for all isosorbide diesters at a concentration of 10 wt %— corroborated by a decrease in both *T*_g_ and tensile strength—no improvement in ductility was observed at this concentration. The brittle–ductile transition became apparent at a plasticizer concentration of 20 wt % (particularly for IDB and IDC), resulting in a significant enhancement of ductile properties, such as elongation at break and impact strength. Gomez-Caturla et al.^70^ observed this threshold in PLA plasticized with esters of tartaric acid, at a plasticizer content between 10 and 20 wt %. Similar findings were reported by Barandiaran et al.^37^ in PLA plasticized with esters of cinnamic acid. Murariu et al.^71^ also highlighted the importance of reaching a threshold composition to achieve the brittle–ductile transition in PLA. Recently, Sun et al.^72^ have demonstrated that this threshold highly depends on the chemical structure of the plasticizer. They found this threshold to be 10 wt % for glyceryl tributyrate, while this increased to 20 wt % for glyceryl triacetate (triacetin) or triethyl citrate (TEC). In the present work, the plasticization efficiency of isosorbide diesters of short- to medium-chain-length (C4–C8) fatty acids is impressive at a concentration of 20 wt % with ε_B_ values of 529.1 and 342.0% for PLA/20IDB and PLA/20IDC, respectively (Figure 4c). Another interesting finding is that PLA and its plasticized formulations with IDB and IDC are transparent, indicating a predominantly amorphous structure. In contrast, plasticized samples with IDP are opaque (gray), since due to partial miscibility, IDP crystals are formed at 69.4 °C during the cooling from the melt. These mechanical results are comparable, or even superior to those described with conventional PLA plasticizers such as citrates, oligomers of lactic acid, polyethylene glycol, polypropylene glycol, or epoxidized vegetable oils, among others.^73^

**Figure 4:**
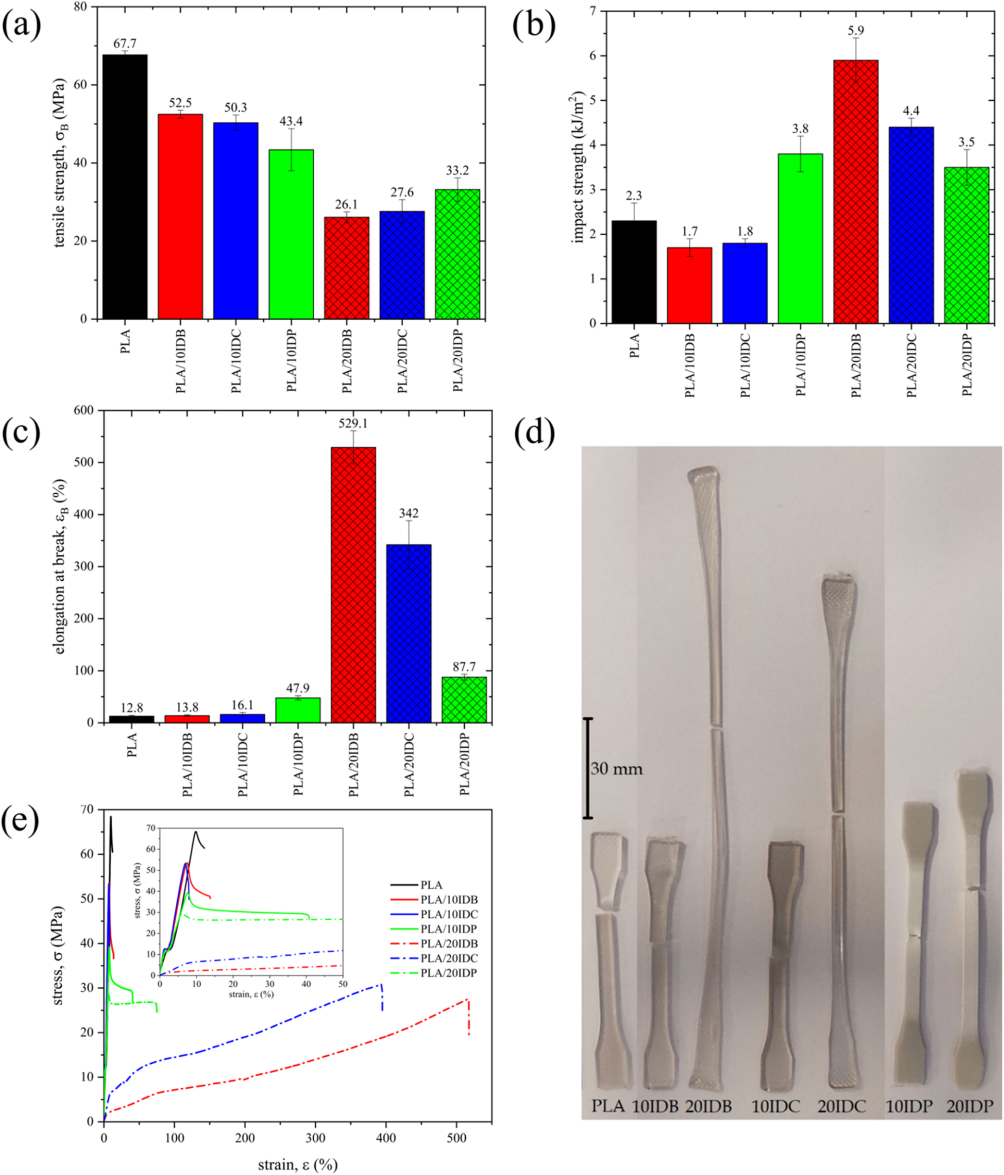
Mechanical properties of neat PLA and plasticized PLA formulations with varying isosorbide diester type and content, (a) tensile strength, σ_B_, (b) elongation at break, ε_B_, (c) Charpy impact strength, (d) visual appearance of specimens after tensile test, and (e) stress (σ)–strain (ε) curves.

The inherent low impact strength of PLA is another factor contributing to its brittleness, with a typical value of 2.3 kJ/m^2^. Similar to ε_B_, the addition of 10 wt % IDB or IDC results in a slight reduction in impact strength, yielding values of 1.7–1.8 kJ/m^2^. However, a significant improvement is observed at higher plasticizer content, with 20 wt % IDB or 20 wt % IDC increasing impact strength to 3.8 and 5.9 kJ/m^2^, respectively (Figure 4b). IDP displays a different behavior, likely due to phase separation. Nevertheless, IDP still enhances impact strength, thus confirming that, even if phase separation occurs, the overall toughness is improved.

Regarding the effect of temperature on mechanical-dynamical properties, Figure 5 presents characteristic DMTA plots (storage, *E*′; loss, *E*″ moduli; and damping factor, tan δ). Note the characteristic DMTA behavior of unplasticized PLA. Below 50 °C, *E*′ remains nearly constant, thus revealing a glassy state (Figure 5a). Between 50 and 70 °C, a pronounced decrease in *E*′ (3 orders of magnitude) is associated with its α-relaxation or glass transition (*T*_g_). The cold crystallization can also be observed by DMTA as an increase in *E*′ beyond *T*_g_.^74^ As expected, the *E*′ vs *T* curves for the plasticized formulations shift to lower temperatures, further confirming the exceptional plasticization efficiency these isosorbide-based plasticizers provide.^75^ This shift results in lower *T*_g_ values, as well as lower *T*_cc_ (Table S3). Figure 5c compares the evolution of the damping factor (tan δ) with the temperature. PLA displays a peak maximum at 64.3 °C, while it decreased at 43–48 °C for plasticized formulations with 20 wt % IDB or IDC. Moreover, regarding plasticized PLA formulations with isosorbide diesters, two evident changes in tan δ peak can be observed (Table S3). On the one hand, the peak height decreases due to the dilution effect, and on the other hand, the full width at half-maximum (fwhm) increases with increasing plasticizer content, which is particularly evident in the case of PLA/20IDC. A similar behavior has been reported by Yeh et al.^76^ in PLA plasticized with triacetin. The increase in FWHW has been attributed to enhanced interactions and contact between PLA and plasticizer molecules, leading to a broader distribution of relaxation times.^77^

**Figure 5:**
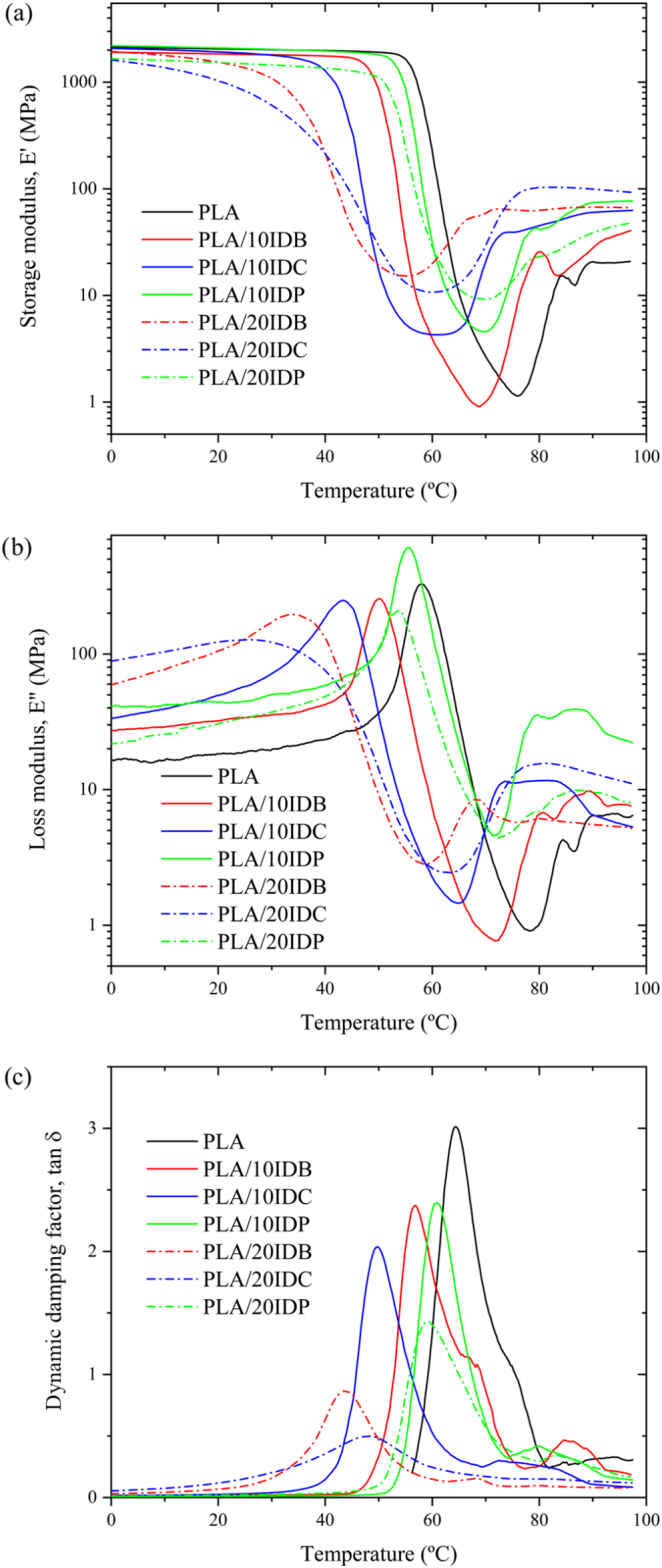
DMTA of neat PLA and plasticized PLA formulations with varying isosorbide diester type and content: (a) storage modulus, *E*′; (b) loss modulus, *E*″; and (c) dynamic damping factor, tan δ.

The FESEM images of the fractured samples after impact testing (Figure 6) further support the previously discussed findings.

**Figure 6:**
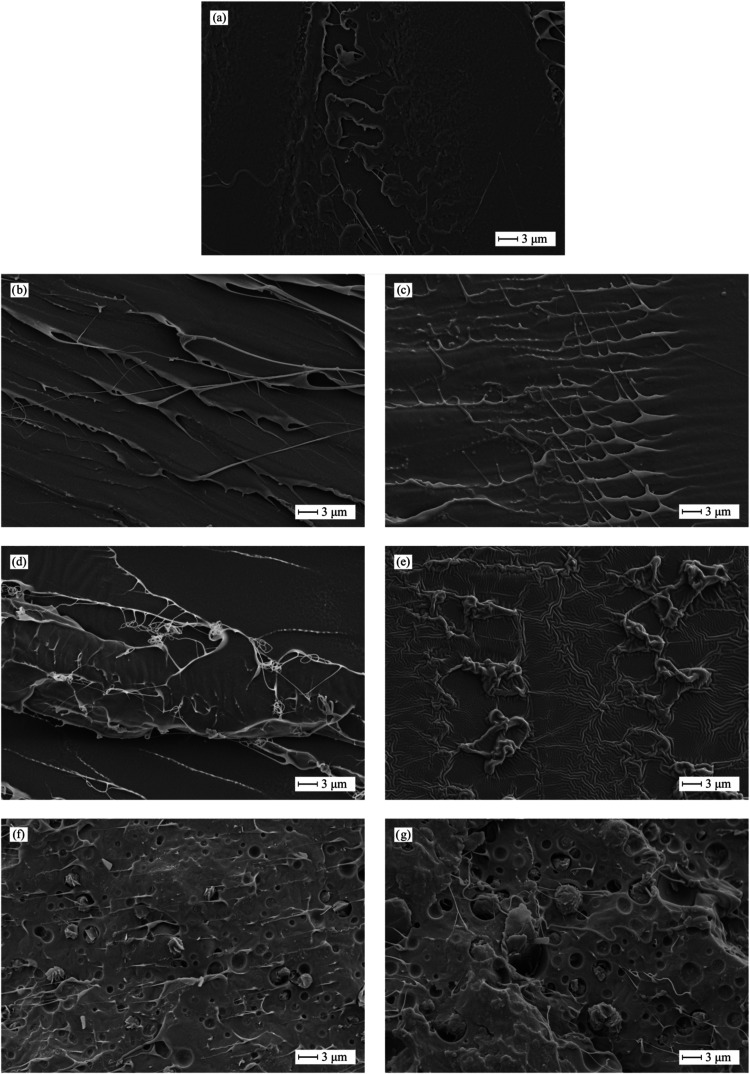
FESEM images (2000×) of the fractured specimens (impact test) of neat PLA and plasticized PLA formulations with varying isosorbide diester type and content: (a) PLA, (b) PLA/10IDB, (c) PLA/20IDB, (d) PLA/10IDC, (e) PLA/20IDC, (f) PLA/10IDP, and (g) PLA/20IDP.

As observed in Figure 6a, PLA exhibits a characteristic brittle fracture displaying smooth, flat surfaces with low roughness.^78^ In contrast, plasticization with IDB results in a homogeneous phase, indicative of good miscibility, as predicted by the Hansen solubility parameters, with IDP showing the lowest RED value of 0.48. Moreover, evidence of plastic deformation is visible in the form of wavy regions and the presence of filament-like structures.^79^ As expected, these plastic deformation features can be clearly seen in PLA/20IDB (Figure 6c), which exhibits an ε_B_ of 529.1%. In contrast, these wavy regions are less pronounced in PLA/10IDB, with just 13.8% ε_B_. Similar behavior can be seen in PLA plasticized with IDC. PLA/10IDC (Figure 6d) shows a rather brittle fracture surface (smooth and flat regions combined with scarce wavy regions and filaments) according to the low ε_B_ of 16.1%, compared to PLA (12.8%). Conversely, PLA/20IDC exhibits a ductile fracture morphology with a wrinkled topography resulting from microdeformations during fracture (Figure 6e). Regarding PLA-IDP blends, a clear phase separation phenomenon can be observed. Despite the partial miscibility and some visible signs of plastic deformation (filament-like structures and rough surface), it is worth noting a biphasic structure with a PLA-rich matrix and spherical IDP particles and crystals finely embedded within the PLA matrix (Figure 6f,6g). This particular morphology significantly contributes to enhancing toughness. The PLA-rich phase, acting as the matrix, shows signs of plastic deformation, suggesting some IDB molecules have entered into the amorphous regions of PLA, thereby improving its ductility, as confirmed by mechanical characterization. Additionally, the island-in-the-sea morphology, characterized by a PLA-rich matrix containing embedded spherical IDB crystals, offers a similar toughening effect. This resembles the behavior of high-impact polystyrene (HIPS), where immiscible butadiene rubber microspheres dispersed within the polystyrene (PS) matrix enhance its toughness.^80^ This morphology is consistent with previous findings and the Flory–Huggins interaction parameter (χ). Mena-Prado et al.^81^ observed phase separation in PLA plasticized with citric acid esters of mono- and diglycerides. Despite that phase separation occurred, they reported a noticeable increase in elongation at break up to 42.5%. Garcia-Garcia et al.^82^ also observed phase separation in PLA plasticized with epoxidized karanja oil beyond 5 wt %, and FESEM study corroborated this by the presence of spherical regions related to excess plasticizer. Therefore, it can be concluded that the fatty acid chain length plays a key role in miscibility. Isosorbide esters with short to medium chain lengths (C4 and C8) lead to exceptional miscibility, while its esters with a long-chain fatty acid (C16) show phase separation. FESEM images at lower (500×) and higher (3000×) can be seen in Figures S8 and S9, respectively, further confirming the above-mentioned findings.

### Disintegration in Compost Soil

Environmental issues related to biodegradation must also be considered to ensure a low environmental impact and sustainable PLA formulations. Isosorbide diesters are fully biobased, but it is also important to assess their effect on disintegration when incorporated into PLA formulations, so they fulfill the “double green” criteria, which means, fully biobased and biodegradable,^83^ along with the technical requirements they must provide. Figure 7 illustrates the evolution of the mass loss during disintegration of plasticized PLA formulations with isosorbide diesters in composting environment as well as the visual appearance of samples during this test. PLA undergoes disintegration within approximately 35 days. The addition of isosorbide diesters significantly influences the disintegration rate, with formulations containing short- (C4) and medium-chain-length (C8) fatty acids exhibiting a higher disintegration rate compared with PLA. After an incubation period of 7 days, disintegration proceeds faster than in neat PLA. In fact, the mass loss is <80% after 21 days, while PLA needs 35 days to reach a similar disintegration rate.

**Figure 7:**
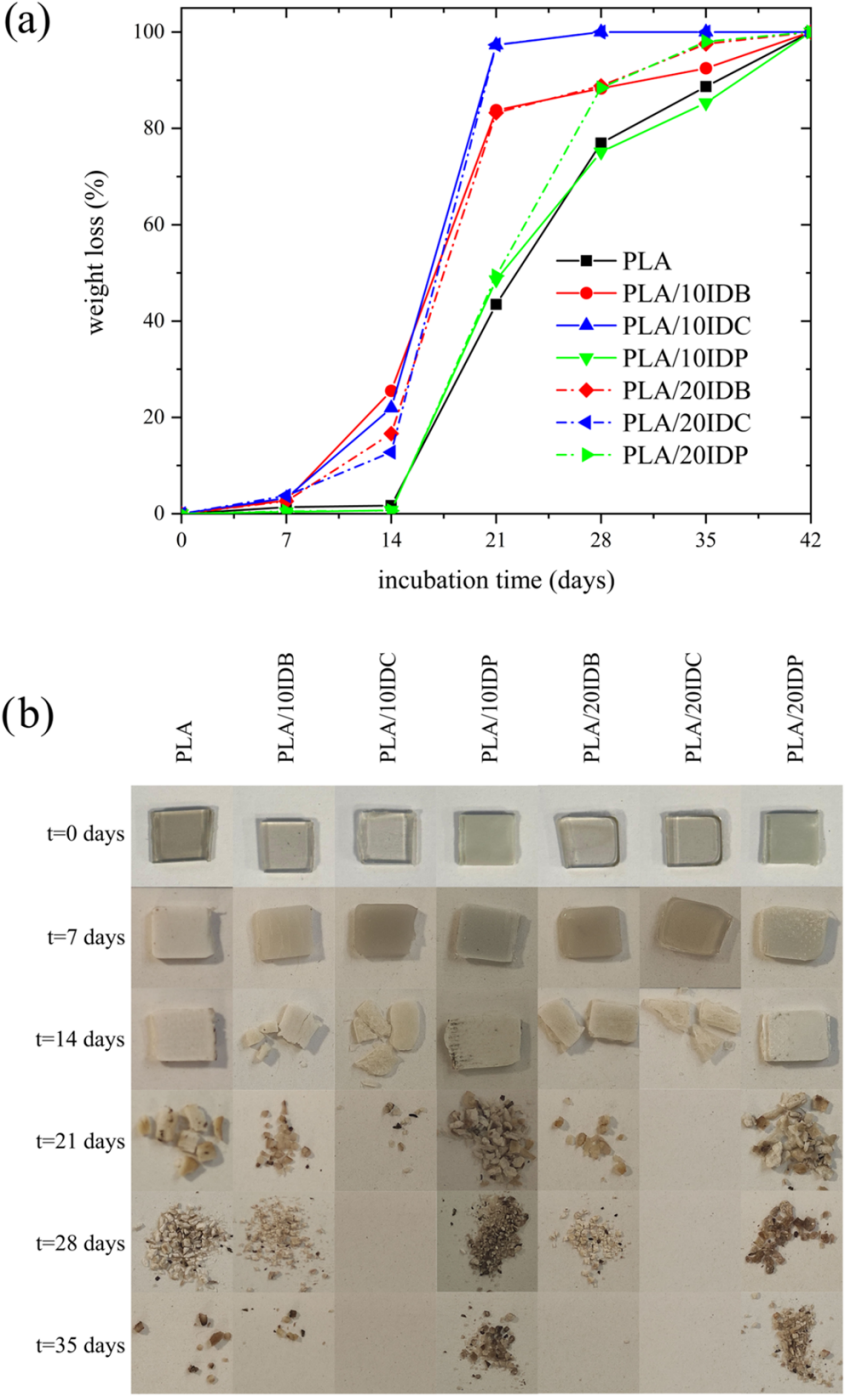
(a) Mass loss evolution and (b) visual appearance with increasing incubation time corresponding to neat PLA and plasticized PLA formulations with varying isosorbide diester type and loading.

It is important to bear in mind that biodegradation processes carried out by microorganisms predominantly occur in amorphous regions. As previously discussed, the injection-molded samples are mainly amorphous as this PLA grade cannot develop high crystallinity during the cooling from the melt.^24^ In general, as the fatty acid chain length increases, the disintegration becomes slower,^84^ but crystallinity also plays an important role in disintegration as crystalline regions are less accessible than amorphous regions.^66^ In the case of plasticized PLA with IDB, although IDB contains short-chain-length fatty acids (C4) and is more prone to biodegradation,^85^ as XRD has revealed (Figure S6), it leads to slightly higher crystallinity than IDC- and IDP-plasticized PLA samples.

Thus, the overall effect on biodegradation is a disintegration rate between that observed for IDC-plasticized materials and PLA. The maximum degradation rate is obtained with IDC, which contains medium-chain-length (C8) fatty acids, with excellent combination of low crystallinity and balanced hydrocarbon backbone length. It is worth noting that disintegration primarily proceeds in the amorphous regions. All PLA materials plasticized with isosorbide diesters are predominantly amorphous, as confirmed by differential scanning calorimetry, which is a crucial factor in the disintegration process. Additionally, the free volume plays a significant role in promoting hydrolysis, thereby enhancing disintegration. Given that all of the obtained materials were mostly amorphous, the chain length of the fatty acid moiety is relevant. In PLA plasticized with IDB (containing C4 fatty acid), the free volume is relatively low due to the smaller molecular size. Conversely, in PLA plasticized with IDC (containing C8 fatty acid), the larger molecules increase the free volume, which, in turn, accelerates disintegration. For IDP (containing C16 fatty acids), phase separation leads to crystalline IDP regions that hinder the disintegration process.

## Conclusions

The plasticization efficiency on polylactide (PLA) of various isosorbide diesters synthesized with fatty acids of varying chain length (C4, C8, and C16), namely, isosorbide dibutyrate (IDB), dicaprylate (IDC), and dipalmitate (IDP), was systematically evaluated. Isosorbide esters with short- (C4) and medium-chain-length (C8) fatty acids exhibit exceptional miscibility with PLA, as predicted by the Hansen solubility parameters (δ) and the Flory–Huggins interaction parameter (χ). This prediction was confirmed experimentally through a noticeable decrease in *T*_g_. Additionally, FESEM analysis revealed monophasic structure for both IDB and IDC, which showed a remarkable increase in elongation at break from 12.8% (PLA) up to 529.1 and 342.0%, respectively. Conversely, PLA plasticized with IDP exhibited partial miscibility. This phase separation was confirmed by a melting peak at 69.4 °C corresponding to IDP crystalline domains and a dual-phase morphology observed by FESEM. This limited miscibility of IDP in PLA resulted in a moderate decrease in *T*_g_, with a subsequent modest increase in ductile properties. The fatty acid chain length greatly influences disintegration in compost soil of PLA-based formulations. Overall, isosorbide diesters emerge as “double green” plasticizers (both biobased and biodegradable) capable of replacing petroleum-derived plasticizers in PLA formulations. Since both PLA and isosorbide are starch-derived, this research aligns with the principles of starch-based biorefineries. Such an approach promotes the development of cost-effective and environmentally sustainable routes for producing both industrial polymers and additives, offering a feasible alternative to petroleum-based plastics. By using starch as a renewable raw resource, this strategy contributes to reducing environmental impact while supporting the advancement of greener solutions in the polymer industry.

## Supplementary Material



## Notes

Physical and spectroscopic data of the synthesized isosorbide diesters, ^13^C NMR spectra of the synthesized isosorbide diesters, FTIR characterization of PLA-isosorbide diester blends, approach to polymer-plasticizer miscibility by the Hansen solubility parameters (δ), and the interaction parameter (χ) of the Flory–Huggins lattice theory, X-ray diffraction spectroscopy characterization, thermogravimetric analysis (TGA) curves, dynamic mechanical thermal analysis (DMTA) data, and field emission scanning electron microscopy (FESEM) images at different magnifications (PDF)
