# Supplementary material for: Sustainable Approach to Overcome Polylactide Brittleness with Biobased Esters of Isosorbide and Fatty Acids
Source: ACS Sustain Chem Eng. 2025 May 16;13(21):7962–74. doi: 10.1021/acssuschemeng.5c01601 (PMC12135067; doi:10.1021/acssuschemeng.5c01601)
Supplement: Supplementary file 1 [file sc5c01601_si_001.pdf]

Supporting Information

Sustainable approach to overcome polylactide brittleness with biobased esters of isosorbide and fatty acids

Mario Miranda-Pinzon†, Jaume Gomez-Caturla†, Juan Ivorra-Martinez†,

Nestor Guijarro‡, Xavier Marset‡□, Rafael Balart†,[*]

† Instituto Universitario de Investigación de Tecnología de Materiales (IUITM), Universitat Politècnica de València (UPV), Plaza Ferrándiz y Carbonell 1, 03801, Alcoy, Alicante, Spain. M.M-P.: mmirpin@epsa.upv.es; J.G-C.: jaugoca@epsa.upv.es; J.I-M.: juaivmar@doctor.upv.es; R.B.: rbalart@mcm.upv.es.

‡ Instituto de Electroquímica, Universidad de Alicante, Apdo. 99, E-03080 Alicante, Spain. N.G.: nestor.guijarro@ua.es

□ Instituto de Síntesis Orgánica (ISO), Universidad de Alicante, Apdo. 99, E-03080 Alicante, Spain. X.M.: xavier.marset@ua.es

It contains 19 pages, 9 figures, and 3 tables.

[*] **Corresponding author:** R. Balart - rbalart@mcm.upv.es
Instituto Universitario de Investigación de Tecnología de Materiales (IUITM)
Universitat Politècnica de València (UPV)
Plaza Ferrándiz y Carbonell 1, 03801, Alcoy, Alicante, Spain.
Tel.: +34 96 652 84 00

S1. PHYSICAL AND SPECTROSCOPIC DATA OF THE SYNTHESIZED ISOSORBIDE DIESTERS.

Isosorbide-2,5-dibutyrate (IDB). Obtained from isosorbide and butyryl chloride using general procedure A. Spectroscopic data matched previous literature reports.[1] Yellow oil. ^{1}H NMR (400 MHz, $CDCl_3$) δ 5.19 – 5.08 (m, 2H), 4.79 (t, *J* = 5.0 Hz, 1H), 4.44 (dt, *J* = 4.7, 0.9 Hz, 1H), 3.98 – 3.87 (m, 3H), 3.76 (dd, *J* = 9.8, 5.5 Hz, 1H), 2.31 (t, *J* = 7.6 Hz, 2H), 2.26 (t, *J* = 7.4 Hz, 2H), 1.68 – 1.55 (m, 4H), 0.95 – 0.87 (m, 6H). ^{13}C NMR (101 MHz, $CDCl_3$) δ 173.0, 172.7, 86.0, 80.8, 77.90, 73.8, 73.5, 70.4, 36.0, 35.9, 18.41, 18.38, 13.64, 13.62. IR (ATR): *v* = 2965, 2937, 2877, 1735, 1461, 1365, 1251, 1170, 1093, 977 cm^{-1}. MS (EI): 286 (M^+, 9), 111 (11), 110 (30), 69 (19), 43 (33).

Isosorbide-2,5-dicaprylate (IDC). Obtained from isosorbide and capryloyl chloride using general procedure A. Spectroscopic data matched previous literature reports.[1] Yellow oil. ^{1}H NMR (400 MHz, $CDCl_3$) δ 5.18 (d, J = 3.5 Hz, 1H), 5.14 (q, *J* = 5.7 Hz, 1H), 4.81 (t, *J* = 5.0 Hz, 1H), 4.46 (d, *J* = 4.7 Hz, 1H), 4.01 – 3.90 (m, 3H), 3.78 (dd, *J* = 9.7, 5.4 Hz, 1H), 2.35 (t, *J* = 7.5 Hz, 2H), 2.29 (t, *J* = 7.5 Hz, 2H), 1.69 – 1.54 (m, 4H), 1.35-1.20 (m, 16 H), 0.87 (t, *J* = 6.9 Hz, 6H). ^{13}C NMR (101 MHz, $CDCl_3$) δ 173.2, 172.9, 86.0, 80.7, 77.9, 73.7, 73.5, 70.3, 34.2, 34.0, 31.63, 31.61, 29.0, 28.89, 28.86, 24.9, 24.8, 22.6, 14.0. IR (ATR): *v* = 2954, 2925, 2856, 1739, 1461, 1375, 1160, 1099, 977 cm^{-1}. MS (EI): 398 (M^+, 1), 254 (17), 128 (12), 127 (79), 111 (13), 69 (35), 57 (47), 55 (14), 41 (10).

Isosorbide-2,5-dipalmitate (IDP). Obtained from isosorbide and palmitoyl chloride using general procedure A. Spectroscopic data matched previous literature reports.[1] White solid, m.p. = 68-69 ºC (EtOH). ^{1}H NMR (400 MHz, $CDCl_3$) δ 5.18 (d, *J* = 3.3 Hz, 1H), 5.14 (q, *J* = 5.6 Hz, 1H), 4.82 (t, *J* = 5.0 Hz, 1H), 4.46 (d, *J* = 4.7 Hz, 1H), 4.01 – 3.91 (m, 3H), 3.78 (dd, *J* = 9.8, 5.4 Hz, 1H), 2.36 (dd, *J* = 8.2, 7.0 Hz, 2H), 2.30 (t, *J* = 7.5 Hz, 2H), 1.67 – 1.54 (m, 3H), 1.31 – 1.22 (m, 52H), 0.87 (t, *J* = 6.8 Hz, 6H). ^{13}C NMR (101 MHz, $CDCl_3$) δ 173.4, 173.0, 86.1, 80.9, 78.0, 73.9, 73.6, 70.5, 34.3, 34.1, 32.1, 29.83, 29.79, 29.7, 29.6, 29.5, 29.40, 29.36, 29.2, 25.01, 24.98, 22.8, 14.3. IR (ATR): *v* = 2954, 2917, 2848, 1729, 1467, 1168, 1097, 721 cm^{-1}. HRMS (EI) calcd. for $C_{38}H_{70}O_6$ (M^+): 622.5172, found: 622.5169.

S2. ^{13}C NMR SPECTRA OF THE SYNTHESIZED ISOSORBIDE DIESTERS.

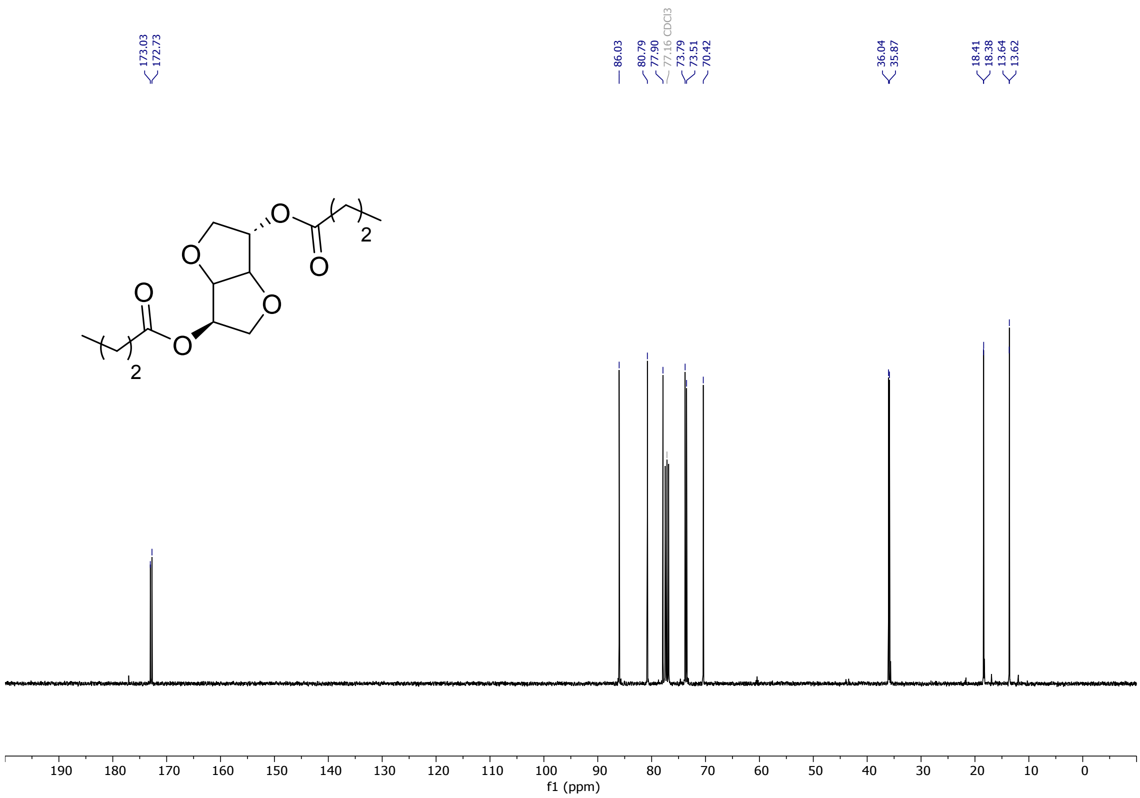

Figure S1.^{13}C NMR ($CDCl_3$, 101 MHz) of synthesized isosorbide-2,5-dibutyrate (IDB).

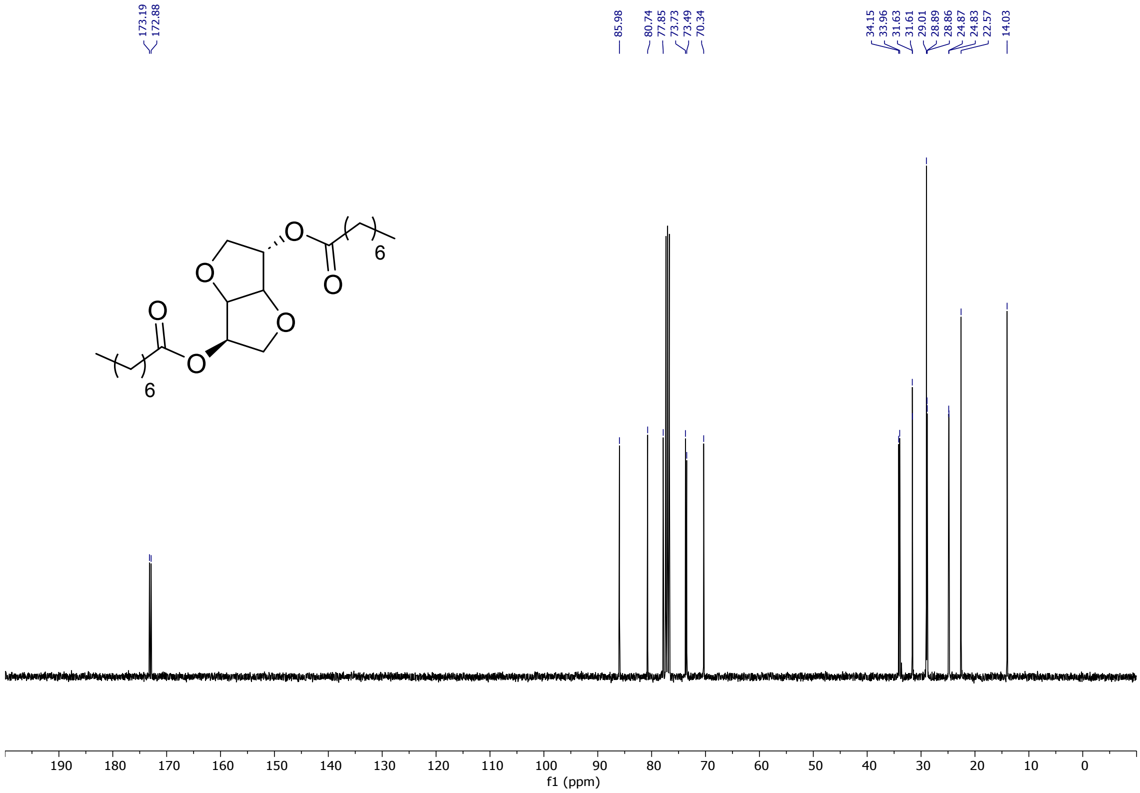

Figure S2.^{13}C NMR ($CDCl_3$, 101 MHz) of synthesized isosorbide-2,5-dicaprylate (IDC).

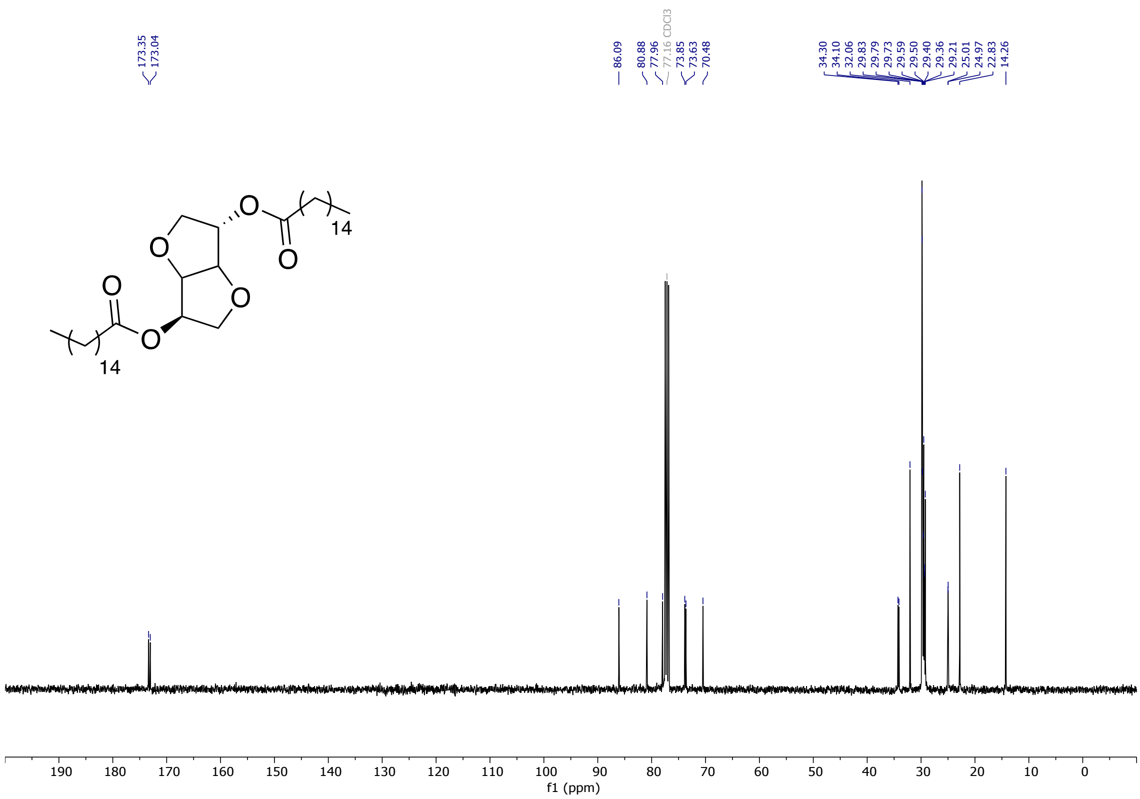

Figure S3. ^{13}C NMR ($CDCl_3$, 101 MHz) of synthesized isosorbide-2,5-dipalmitate (IDP).

S3. FTIR CHARACTERIZATION OF PLA-ISOSORBIDE DIESTER BLENDS.

FTIR further corroborated the presence of the different synthesized isosorbide esters within the PLA blends as depicted in **Figure S4**. Given that PLA constitutes the primary component of the plasticized formulations, its characteristic peak/bands are prominently visible. Notably, the absorption bands at 1751 cm^{-1} and 1180 cm^{-1} correspond to the stretching vibration of the –C=O, and –C–O contained in the ester groups. Additionally, an absorption peak at 2994 cm^{-1} is attributed to the asymmetric and symmetric stretching vibrations of the aliphatic C–H bonds,[2] consistent with typical absorption bands in the 2990 – 2940 cm^{-1} range. The bending (scissoring or deformation) vibration of the methyl group (–CH_3) appears at 1450 cm^{-1}, whereas the bending vibrations of the C–H can be observed within the 1380 – 1360 cm^{-1} range. The absorption peak at 868 cm^{-1}, typical of the crystalline regions of PLA, is also present. Note the spectra of the plasticized formulations barely changed with respect to that of the PLA.

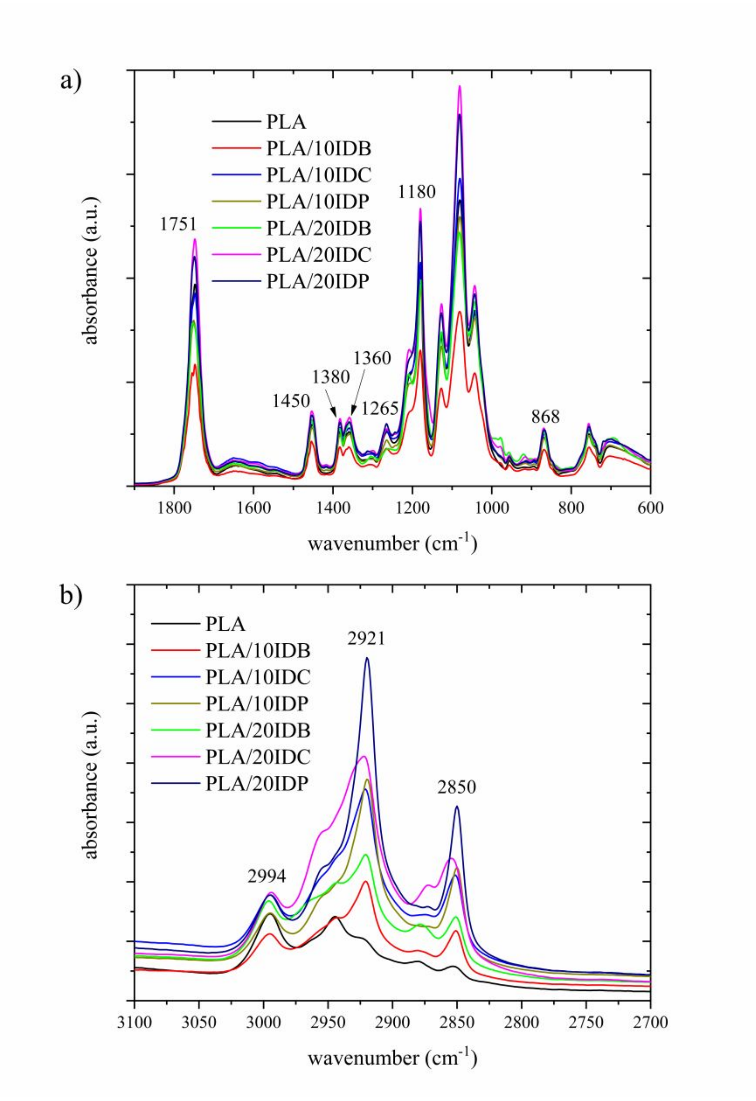

Figure S4. FTIR spectra in different wavenumber regions, a) 1700 – 600 cm^{-1}, and b) 3100 – 2700 cm^{-1}, corresponding to neat PLA and plasticized PLA formulations with varying isosorbide diester type and content.

This is not unexpected given PLA is the major component. However, specific absorption bands attributed to isosorbide diesters can be seen. The most pronounced spectral changes occur in the 2960 – 2840 cm^{-1} absorption region, corresponding to the increasing presence of $–CH_2–$ groups in the synthesized isosorbide diesters, following the trend IDP > IDC > IDB. In particular, the asymmetric C–H stretching vibration appears at 2921 cm^{-1}, with the peak intensity increasing proportionally with the number of $–CH_2–$ groups. Similar tendency is observed for the C–H

symmetric stretching vibration located at 2850 cm^{-1}, with the maximum peak intensity for the plasticized PLA formulation containing 20 wt.% IDP, as expected (**Figure S4b**). Other peaks related to $–CH_2–$ groups include the C–H bending vibrations, such as scissoring, wagging, and twisting, located at 1454 cm^{-1}, 1357 cm^{-1}, and 1265 cm^{-1}, respectively but as PLA also shows these peaks, they overlap.

S4. THEORETICAL BACKGROUND OF POLYMER-PLASTICIZER MISCIBILITY.

S4.1. The solubility parameter, δ.

The cohesive energy, E_{coh} of a substance can be defined as the increment in internal energy, U, per unit mole in the particular case all intermolecular forces/interactions are removed. The cohesive energy density, echo can be determined as indicated in **Eq. S1**.

$$e_{coh} = \frac{E_{coh}}{V} \qquad \textbf{Eq. S1}$$

The solubility parameter is defined as shown in **Eq. S2**.

$$\delta = {e_{coh}}^{1/2} = \left(\frac{E_{coh}}{V}\right)^{1/2} \text{ with units in } (J/cm^3)^{1/2} \text{ or } (MJ/m^3)^{1/2} \qquad \textbf{Eq. S2}$$

For low molecular weight substances, E_{coh} can be obtained by **Eq. S3**.

$$E_{coh} = \Delta U_{vap} = \Delta H_{vap} - p\Delta V \approx \Delta_{vap} - RT \qquad \textbf{Eq. S3}$$

As polymers cannot be directly evaporated, there are several experimental methods to determinate their cohesive energy such as comparative swelling, dissolution experiments, among others. Nevertheless, these experimental methods are time-consuming and despite they provide reliable information, other theoretical approaches are often employed as screening tools to assess

polymer-solvent (or plasticizer) miscibility. Several group contribution methods have been proposed as a simple way to predict polymer-plasticizer miscibility or phase separation.

The solubility parameter includes three different contributions, one corresponding to the dispersive forces, denoted as δ_d, one related to the polar forces, δ_p, and a third component related to the hydrogen bonding interactions, δ_H. The relationship between δ and its contributions is shown in **Eq. S4**.

$$\delta^2 = \delta_d^2 + \delta_p^2 + \delta_H^2 \qquad \textbf{Eq. S4}$$

The different components can be obtained following **Eq. S5** to **S7**,[3] with the molar attraction constants of each structural chemical group (i), namely F_{di}, and F_{pi}, for the dispersive and polar contributions, respectively, and the molar volume, V_m, which results from the ratio of the molecular weight to the density. Regarding to the hydrogen bonding contribution, δ_H, as indicated by Hansen, instead of the molar attraction constants, the cohesive energy, E_{Hi}, is constant for a particular structural group and then, it can be used to determine δ_H as indicated in **Eq. S7**.

$$\delta_d = \frac{\sum F_{di}}{V_m} \qquad \textbf{Eq. S5}$$

$$\delta_p = \frac{\sqrt{\sum F_{pi}^2}}{V_m} \qquad \textbf{Eq. S6}$$

$$\delta_H = \sqrt{\frac{\sum E_{Hi}}{V_m}} \qquad \textbf{Eq. S7}$$

To visualize the different contributions of the solubility parameter and compare them, a 2D plot of the solubility parameters has been used (Bagley's plot), with δ_v (which includes de polar and dispersive contributions), calculated as indicated in **Eq. S8**.

$$\delta_v = \sqrt{\delta_d^2 + \delta_p^2} \quad \textbf{Eq. S8}$$

S4.2. The Flory-Huggins interaction parameter, χ.

The Flory-Huggins lattice theory considers a fluid model where solvent molecules occupy lattice sites, while polymer chains are connected along neighboring sites.[4, 5] The Gibbs free energy of mixing contains an enthalpic and an entropic contribution as shown in **Eq. S9**.

$$\Delta G_M = \Delta H_M - T\Delta S_M = nRT\left[\frac{\varphi}{x}\ln\varphi + (1-\varphi)\ln(1-\varphi) + \chi\varphi(1-\varphi)\right] \quad \textbf{Eq. S9}$$

Where n represents the number of lattice mole, φ stands for the polymer volume fraction, (1-φ) represents the solvent (plasticizer) volume fraction, x is the degree of polymerization, and χ is the so-called Flory-Huggins interaction parameter. Rearranging terms in **Eq. S9**, the enthalpic, and entropic contributions are shown in **Eq. S10** and **Eq. S11** respectively.

$$\Delta H_M = nRT\chi\varphi(1-\varphi) \quad \textbf{Eq. S10}$$

$$T\Delta S_M = nRT\left[\frac{\varphi}{x}\ln\varphi + (1-\varphi)\ln(1-\varphi)\right] \quad \textbf{Eq. S11}$$

The interaction parameter, χ, of the Flory-Hugging theory gives additional and valuable information. χ plays a critical role in predicting the thermodynamics of polymer mixtures and phase behaviour of a polymer-solvent system. This also includes an entropic and a enthalpic contribution as seen in **Eq. S12**. This parameter has been widely used to assess the miscibility between a polymer and a solvent (or plasticizer),[6] and provides additional information about phase separation in plasticized polymers blends.

$$\chi = \chi_S + \chi_H \quad \textbf{Eq. S12}$$

The entropic contribution is a constant comprised between 0.3 and 0.4, but usually, a value of 0.34 is applied for non-polar systems.[7]

With regard to the enthalpic contribution, Hildebrand established the relationship of the enthalpy of mixing with the solubility parameter as shown in **Eq. S13**.

$$\frac{\Delta H_M}{V} = \varphi(1-\varphi)(\delta_2 - \delta_1)^2 \qquad \textbf{Eq. S13}$$

Where the term in the left-side is the enthalpy of mixing per volume, and δ_1, and δ_2 stand for the solubility parameters of the solvent, and polymer, respectively. By combining **Eq. S10** and **Eq. S13**, it is possible relate between the Hansen solubility parameter (δ), and the Flory-Huggins interaction parameter (χ). By changing subscript 1 to plasticizer (IDE), and 2 to polymer (PLA), this expression allows calculating the enthalpic contribution to interaction parameter of PLA and the synthesized isosorbide diesters (IDE), as shown in **Eq. S14**:

$$\chi_H = \frac{\nu_{IDE}}{RT}(\delta_{PLA} - \delta_{IDE})^2 \qquad \textbf{Eq. S14}$$

Therefore, the global interaction parameter, χ, can be obtained by **Eq. S15**.

$$\chi = 0.34 + \frac{\nu_1}{RT}(\delta_{PLA} - \delta_{IDE})^2 \qquad \textbf{Eq. S15}$$

There is a χ critical value to reach the so-called θ solvent condition, and means the polymer-solvent (plasticizer) interactions are equivalent to polymer-polymer interactions, and plasticizer-plasticizer interactions. This depends on the degree of polymerization (x) of the considered polymer, and can be obtained following **Eq. S16**.

$$\chi_{crit} = \frac{1}{2} + \frac{1}{\sqrt{x}} + \frac{1}{2x} \qquad \textbf{Eq. S16}$$

Dissolution of high molecular weight polymers in a solvent (or plasticizer) is only possible if $\chi < \chi_{crit}$ (usually taken as 0.5 for high molecular weight polymers).

Table S1, and **Table S2**, contain the thermodinamical parameters of isosorbide esters compared with common PLA plasticizers, and some good PLA solvents, respectively. These tables gather the parameters from the Hansen solubility theory, namely the solubility parameter (δ), and its contributions (δ_d, δ_p, and δ_H), the distance between solubility parameters (R_a), and the relative energy dispersion (RED). Moreover, the interaction parameter from the Flory-Huggins theory, is included at two temperatures, namely at room temperature (χ_{RT}), and processing temperature of PLA at 190 ºC (χ_{PT}).

Table S1. Thermodinamical parameters related to the miscibility of PLA with isosorbide diesters, and some commonly used PLA plasticizers.

**Plasticizer**	δ_d **(MPa$^{1/2}$)**	δ_p **(MPa$^{1/2}$)**	δ_H **(MPa$^{1/2}$)**	δ **(MPa$^{1/2}$)**	R_a **(MPa$^{1/2}$)**	**RED**	χ_{RT}	χ_{PT}
**PLA**	**15.33**	**8.44**	**10.98**	**20.66**	**-**	**-**	**-**	**-**
IDB	16.00	3.81	9.22	18.85	5.13	0.48	0.65	0.54
IDC	16.37	2.47	7.44	18.15	7.24	0.68	1.26	0.93
IDP	16.65	1.45	5.70	17.66	9.15	0.85	2.57	1.77
TEC	16.17	4.06	13.01	21.15	5.11	0.48	0.36	0.36
ATBC	16.02	2.56	8.55	18.33	6.51	0.61	1.18	0.88
TBC	16.03	2.85	10.89	19.59	5.76	0.54	0.50	0.44
ATEC	16.13	3.50	10.00	19.30	5.29	0.49	0.55	0.48
ESO	14.93	1.26	6.07	16.17	8.74	0.82	8.30	5.46
TriAc (TA)	16.21	4.51	10.57	19.87	4.33	0.40	0.39	0.37
TriBut (TB)	15.91	2.89	8.46	18.25	6.20	0.58	1.03	0.78
PEG400	17.31	3.80	13.50	22.27	6.60	0.62	0.71	0.58
PEG1000	17.82	2.38	11.21	21.18	7.85	0.73	0.43	0.40
LA	16.64	8.76	20.07	27.50	9.46	0.88	1.75	1.25
OLA-OH	14.60	1.93	11.52	18.70	6.69	0.63	1.87	1.32
DEHA/DOA	15.97	1.73	5.91	17.12	8.51	0.80	2.36	1.64
DBA	16.09	2.58	7.22	17.82	7.12	0.67	1.21	0.90
DEHP/DOP	16.70	1.78	5.96	17.82	8.78	0.82	1.62	1.16
DBP	17.01	2.65	7.27	18.69	7.66	0.72	0.76	0.61
TOTM	21.35	1.57	6.17	22.28	14.67	1.37	0.92	0.71

IDB-isosorbide dibutyrate; **IDC**-isosorbide dicaprylate; **IDP**-isosorbide dipalmitate; **TEC**-triethyl citrate; **ATBC**-acetyltributyl citrate; **TBC**-tributyl citrate; **ATEC**-acetyltriethyl citrate; **ESO**-epoxidized soybean oil; **TAc**-triacetin; **TBu**-tributyrin; **PEG400**, **PEG1000**-polyethyleneglycol; **LA**-lactic acid; **OLA-OH**-oligomeric lactic acid hydroxyl terminated; **DEHA**-bis(2-ethylhexyl) adipate; **DBA**-dibutyl adipate; **DEHP**-bis(2-ethylhexyl) phthalate; **DBP**-dibutyl phthalate; **TOTM**-trioctyl trimellitate.

Table S2. Thermodinamical parameters related to the miscibility of PLA with common PLA solvents.

**Solvent**	**δ_d ($MPa^{1/2}$)**	**δ_p ($MPa^{1/2}$)**	**δ_H ($MPa^{1/2}$)**	**δ ($MPa^{1/2}$)**	**R_a ($MPa^{1/2}$)**	**RED**	**χ_{RT}**	**χ_{PT}**
**PLA**	**15.33**	**8.44**	**10.98**	**20.66**	**-**	**-**	**-**	**-**
chloroform	17.80	3.10	5.70	18.95	8.99	0.84	0.44	0.40
dichloromethane	18.20	6.30	6.10	20.20	7.83	0.73	0.35	0.34
tetrahydrofuran	16.80	5.70	8.00	19.46	5.00	0.47	0.39	0.37
acetone	15.50	10.40	7.00	19.94	4.45	0.42	0.36	0.35
benzene	18.40	0.00	2.00	18.51	13.77	1.29	0.51	0.45
toluene	18.00	1.40	2.00	18.16	12.60	1.18	0.61	0.51
methanol	15.10	12.30	22.30	29.61	11.97	1.12	1.65	1.19
dimethylformamide	17.40	13.70	11.30	24.86	6.70	0.63	0.89	0.69
hexafluoroisopropanol	17.20	4.50	14.70	23.07	6.58	0.62	0.59	0.50
ethyl lactate	16.00	7.60	12.50	21.68	2.19	0.21	0.39	0.37
propylene carbonate	20.00	18.00	4.10	27.22	15.03	1.40	1.82	1.29
1,4-dioxane	19.00	1.80	7.40	20.47	10.53	0.98	0.34	0.34
N-methyl-2-pyrrolidone	18.00	12.30	7.20	22.96	7.60	0.71	0.55	0.47
methylethyl ketone	17.80	3.10	5.70	18.95	8.99	0.84	0.44	0.40

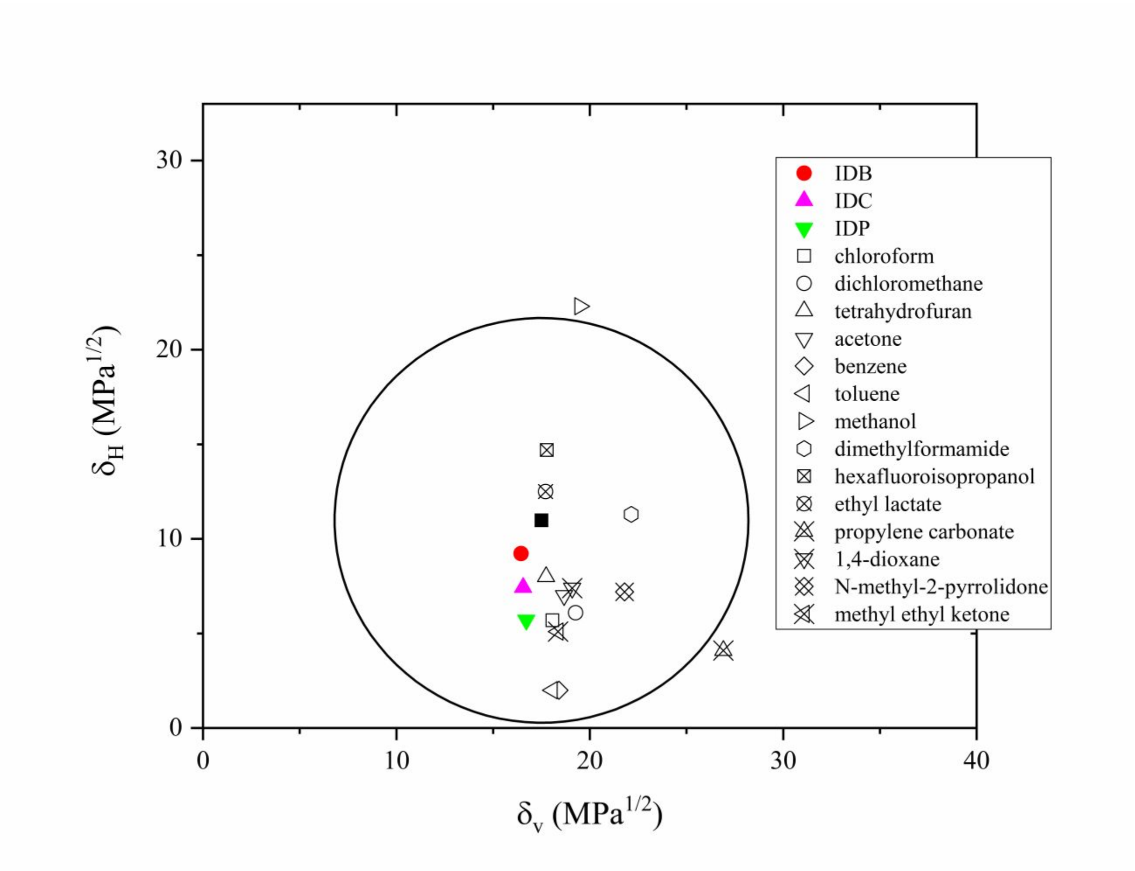

Figure S5. Bagley's solubility diagram for polylactide and the synthesized isosorbide diesters. The plot also includes the location of some common good PLA solvents.

S5. X-RAY DIFFRACTION SPECTROSCOPY CHARACTERIZATION.

The mainly amorphous structure developed by PLA and plasticized PLA formulations with isosorbide diesters was also confirmed by XRD (**Figure S6**).

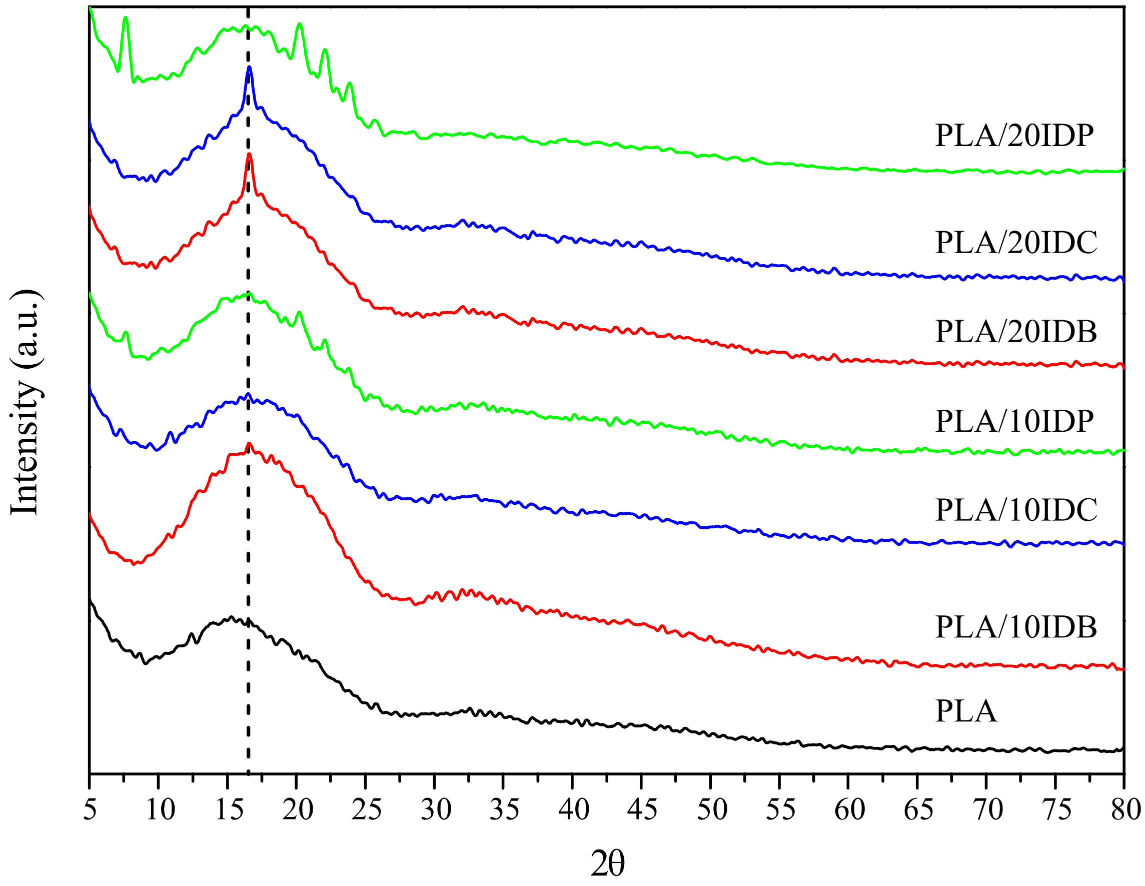

Figure S6. X-ray diffraction patterns corresponding to neat PLA and plasticized PLA formulations with varying isosorbide diester type and content.

The X-ray diffractogram after processing displays a wide band ranging from 10 to 25º, which is typical of the amorphous regions of PLA. Luna et al.[8] observed this behaviour in injection-molded PLA formulations are mainly amorphous after processing (without annealing). A similar XRD pattern was observed for PLA subjected to annealing at 70 ºC, whereas annealing at 80 ºC or higher resulted in the formation of a crystalline structure characterized by a well-defined XRD diffraction peak at $2\theta = 16.79º$ which corresponds to the (200) and (110) diffraction planes of α-type crystals, along with a very small peak at $2\theta = 18.98º$ which corresponds to the (203) plane of the α-phase. None of these diffraction peaks are observed in **Figure S6**, except for the

plasticized PLA formulation containing 20 wt.% IDB and IDC with a clear peak arising at 2θ = 16.74º. This indicates that these diesters enhance the crystallization ability of PLA during cooling. Similar findings have been reported by Gomez-Caturla et al.[9] in plasticized PLA with terpenoid-based plasticizers. Interestingly, plasticized PLA formulations with 10, and 20 wt.% IDP exhibit prominent diffraction peaks at 2θ = 7.7º, 20.3º, 22.1º, and 23.9º which are ascribed to immiscible IDP crystalline domains, as further confirmed by DSC analysis.

S6. THERMOGRAVIMETRIC ANALYSIS (TGA) – THERMAL DEGRADATION.

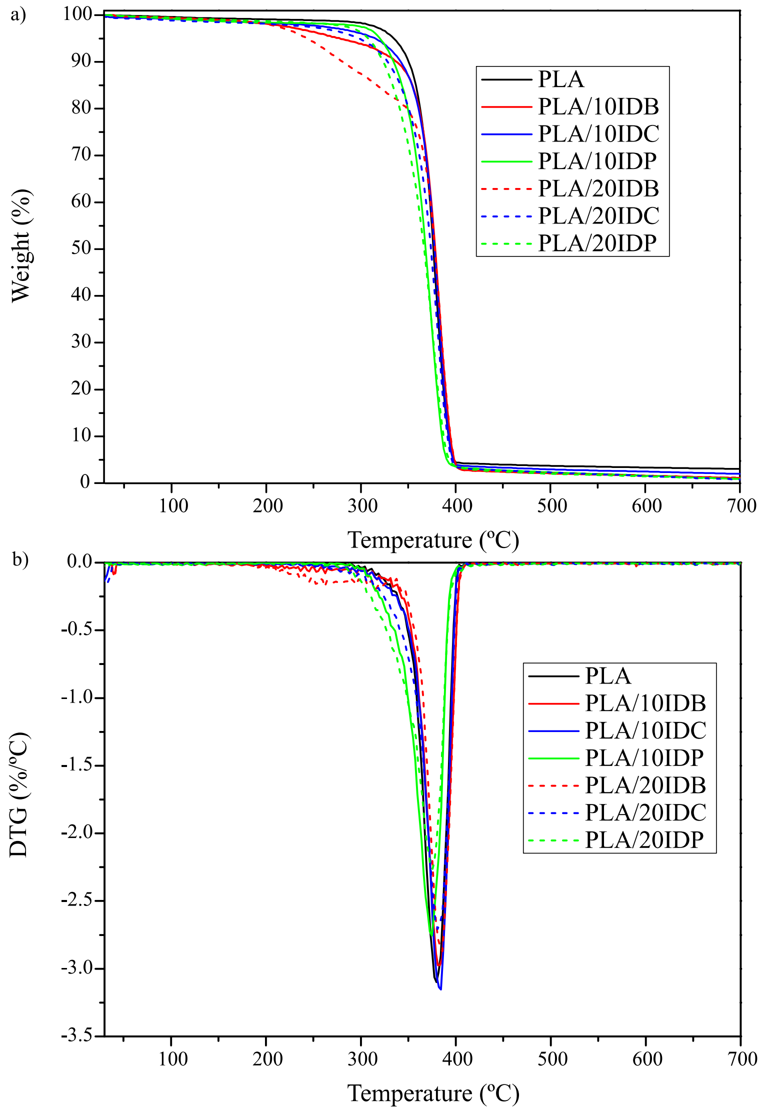

Figure S7. Thermal degradation profiles corresponding to neat PLA and plasticized PLA formulations with varying isosorbide diester type and content, a) sample weight *vs* temperature, and b) first derivative (DTG) *vs* temperature.

S7. DYNAMIC MECHANICAL THERMAL ANALYSIS (DMTA) DATA.

Table S3. Main thermomechanical parameters of neat PLA and plasticized PLA formulations with varying isosorbide diester type and content, obtained dynamical-mechanical thermal analysis (DMTA).

	**Glass transition (GT)**				**Cold crystallization (CC)**	
	**T_{peak}, E" (ºC)**	**T_{peak}, tan δ (ºC)**	**tan δ peak height**	**FWHM tan δ (ºC)**	**T_{onset}, E' (ºC)**	**T_{onset}, E" (ºC)**
PLA	57.8	64.3	3.11	10.1	76.3	79.4
PLA/10IDB	50.2	56.7	2.45	11.4	69.9	73.5
PLA/10IDC	43.4	49.6	2.09	9.5	64.1	64.9
PLA/10IDP	55.4	60.6	2.49	8.9	70.8	72.3
PLA/20IDB	34.2	43.6	0.89	12.5	56.9	59.4
PLA/20IDC	25.2	48.1	0.50	25.5	60.7	64.9
PLA/20IDP	53.5	58.8	1.45	13.5	71.3	72.6

S8. FIELD EMISSION SCANNING ELECTRON MICROSCOPY (FESEM) IMAGES AT DIFFERENT MAGNIFICATIONS.

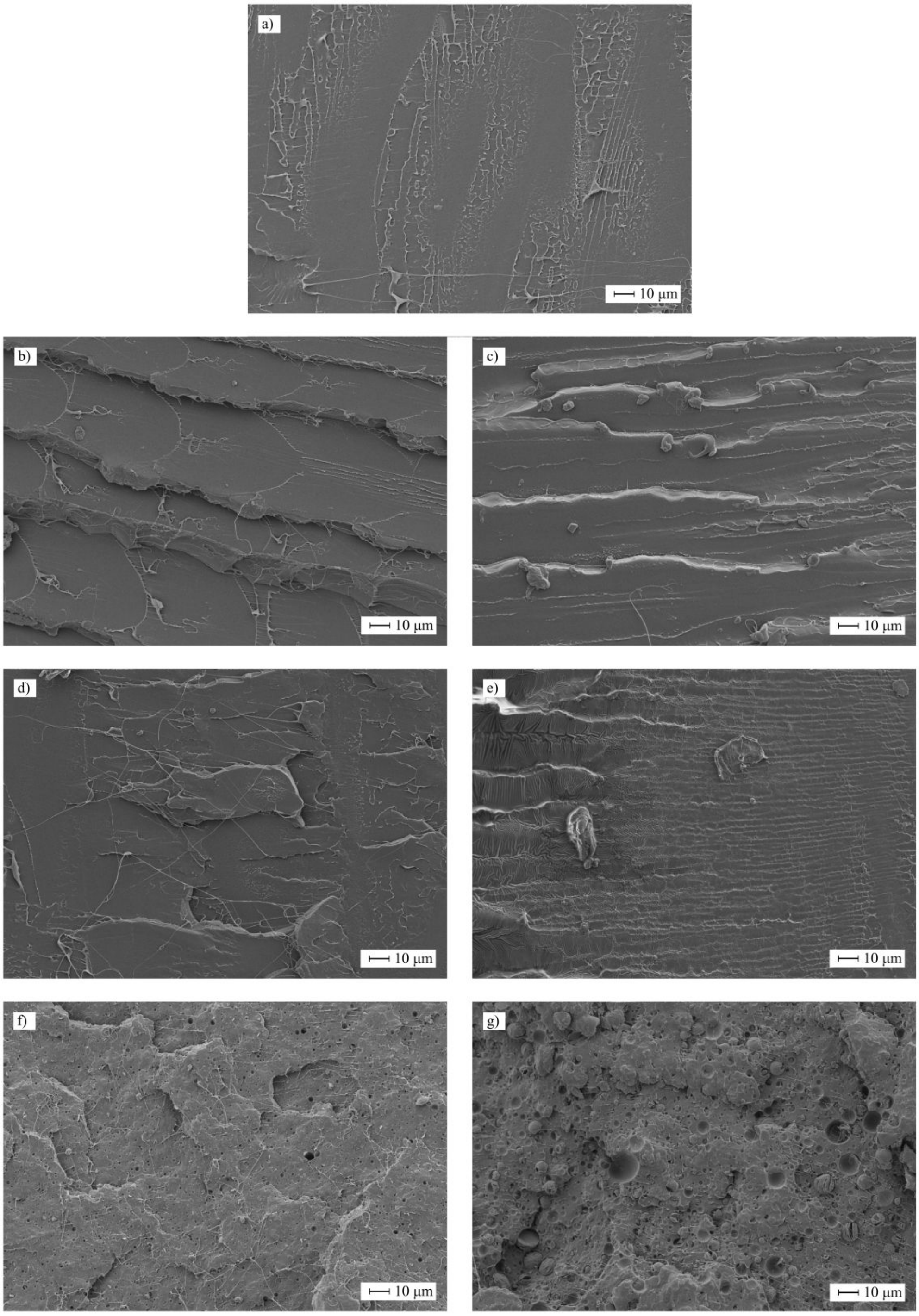

Figure S8. FESEM images (500×) of the fractured specimens (impact test) of neat PLA and plasticized PLA formulations with varying isosorbide diester type and content, a) PLA, b) PLA/10IDB, c) PLA/20IDB, d) PLA/10IDC, e) PLA/20IDC, f) PLA/10IDP, and g) PLA/20IDP.

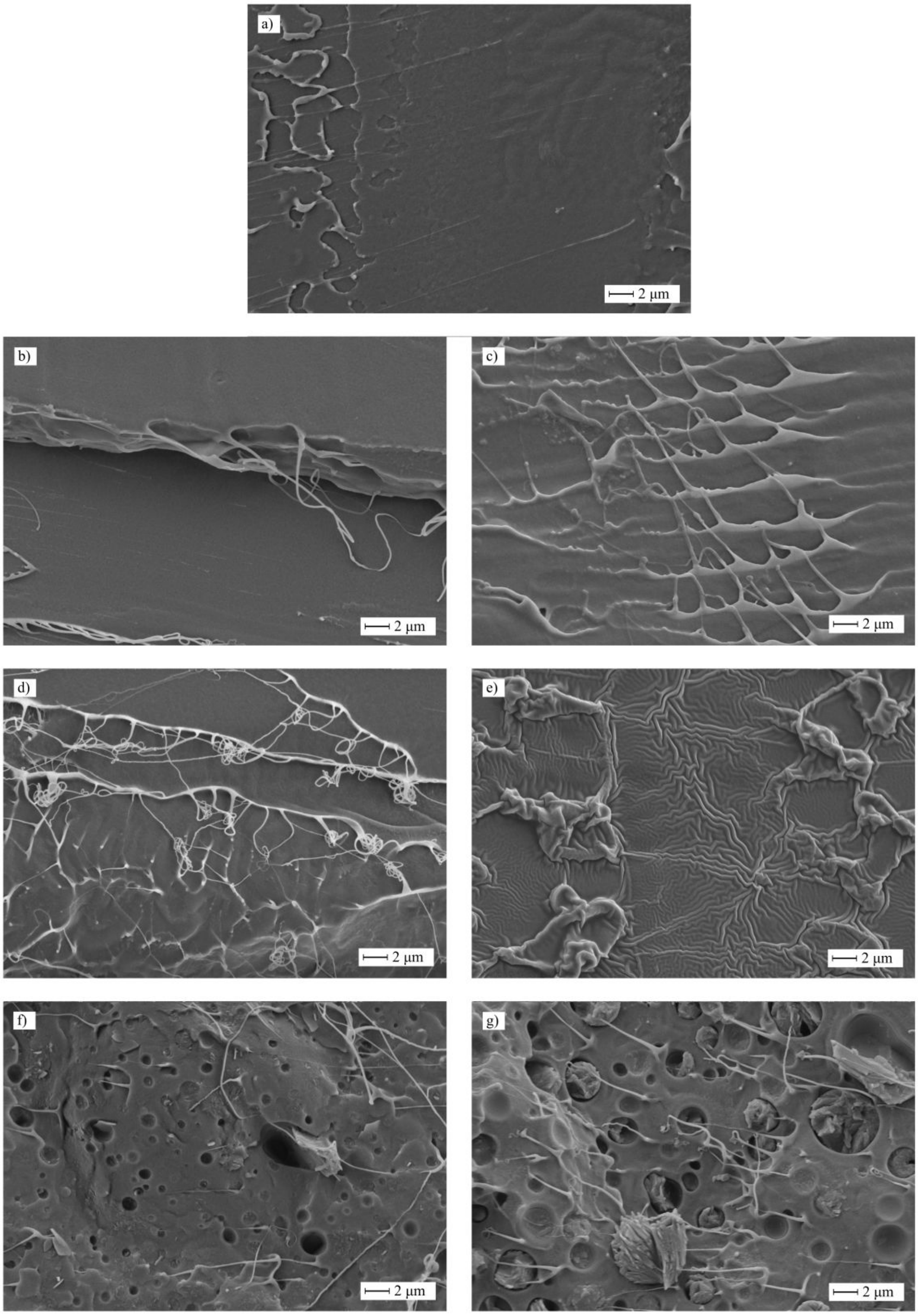

Figure S9. FESEM images (3000×) of the fractured specimens (impact test) of neat PLA and plasticized PLA formulations with varying isosorbide diester type and content, a) PLA, b) PLA/10IDB, c) PLA/20IDB, d) PLA/10IDC, e) PLA/20IDC, f) PLA/10IDP, and g) PLA/20IDP.

REFERENCES.

1. Che, P.H.; Ma, H.; Nie, X.; Yu, W.Q.; Xu, J. Methyl isobutyl ketone-enabled selective dehydration-esterification of sorbitol to isosorbide esters over the H-beta catalyst, *Green Chem.* **2022***,* 24(19), DOI: 7545-7555, 10.1039/d2gc02342c.

2. Beniwal, P.; Guliani, D.; Toor, A.P. Influence of functionalised lignin on strength and antioxidant properties of polylactic acid films, *J. Polym. Res.* **2024***,* 31, 68, DOI: 10.1007/s10965-024-03912-w.

3. van Krevelen, D.W., Properties of Polymers, Elsevier 2012.

4. Flory, P.J. Thermodynamics of high polymer solutions, *J. Chem. Phys.* **1942***,* 10, 51-61, DOI: 10.1063/1.1723621.

5. Huggins, M.L. Theory of solutions of high polymers, *J. Am. Chem. Soc.* **1942***,* 64(7), 1712–1719, DOI: 10.1021/ja01259a068.

6. Murariu, M.; Ferreira, A.D.; Alexandre, M.; Dubois, P. Polylactide (PLA) designed with desired end-use properties: 1. PLA compositions with low molecular weight ester-like plasticizers and related performances, *Polym. Advan. Technol.* **2008***,* 19(6), 636-646, DOI: 10.1002/pat.1131.

7. Pillin, I.; Montrelay, N.; Grohens, Y. Thermo-mechanical characterization of plasticized PLA: Is the miscibility the only significant factor?, *Polymer* **2006***,* 47(13), 4676-4682, DOI: 10.1016/j.polymer.2006.04.013.

8. Luna, C.B.B.; Siqueira, D.D.; Araújo, E.M.; Wellen, R.M.R. Annealing efficacy on PLA. Insights on mechanical, thermomechanical and crystallinirt characters, *Momento-Revista De Fisica* **2021***,* 62, 1-17, DOI: 10.15446/mo.n62.89099.

9. Gomez-Caturla, J.; Tejada-Oliveros, R.; Ivorra-Martinez, J.; Garcia-Sanoguera, D.; Balart, R.; Garcia-Garcia, D. Development and characterization of new environmentally friendly polylactide formulations with terpenoid-based plasticizers with improved ductility, *J. Polym. Environ.* **2024***,* 32(2), 749-762, DOI: 10.1007/s10924-023-03000-y.
